# ﻿A taxonomic revision of *Cenchrus* L. (Poaceae) in Thailand, with lectotypification of *Pennisetummacrostachyum* Benth.

**DOI:** 10.3897/phytokeys.234.106486

**Published:** 2023-09-28

**Authors:** Paweena Wessapak, Chatchai Ngernsaengsaruay, Suthee Duangjai

**Affiliations:** 1 Department of Botany, Faculty of Science, Kasetsart University, Chatuchak, Bangkok 10900, Thailand Kasetsart University Bangkok Thailand; 2 Biodiversity Center, Kasetsart University (BDCKU), Chatuchak, Bangkok 10900, Thailand Kasetsart University Bangkok Thailand; 3 Department of Biology, Faculty of Forestry, Kasetsart University, Chatuchak, Bangkok 10900, Thailand Kasetsart University Bangkok Thailand

**Keywords:** Gramineae, grasses, lectotype, revision, taxonomy

## Abstract

A revision of the genus *Cenchrus* (Poaceae) in Thailand is reported. Seven species, i.e. *C.brownii*, *C.ciliaris*, *C.clandestinus*, *C.echinatus*, *C.pedicellatus*, *C.purpureus* and *C.setosus* are described in this taxonomic treatment. This genus is an exotic species and distributed throughout the floristic regions. All the species have become naturalised to Thailand as a weed and found growing in wastelands, open areas by the roadside, disturbed sites, the edge of rice fields and the edge of deciduous and evergreen forests at elevations between 0 and 2,650 m alt. *Pennisetummacrostachyum*, a synonym of *Cenchruspurpureus* is lectotypified. Detailed morphological descriptions, illustrations and a key to the species are presented, along with information on distributions, habitats, ecology, phenology, vernacular names and specimens examined.

## ﻿Introduction

The Poaceae or grass family, also known as Gramineae, is one of the largest families of flowering plants, consists of approximately 12,000 species in 789 accepted genera and is widely distributed around the world (Soreng et al. 2022). This family is able to adapt to a wide range of habitats as it can survive in different environmental stresses. Grasses are both common and important plants. The world’s human population relies on grasses, which include rice, wheat, oat and maize and are a valuable source of food for humans as they contain fibres, proteins and some nutrients. Most of them have a short life, fast growth and undergo high biomass accumulation. As such, several species are considered as good fodder and a source of nutrients for the livestock. Many species are also used as ornamental plants in landscaping and gardening, for beautifying the lawns. In addition, some grasses are used as raw materials in manufacturing paper, extracting the essential oils and are also used to prevent soil erosion and surface run-off (Chase 1921; Bor 1960; Gould 1968; McIlroy 1972; Oyen and Dung 1999; Obi Reddy et al. 2014; Chavre and Sonawane 2021).

Grasses have various morphological characters. Their reproductive part consists of a small spikelet which is a significant characteristic used in their identification. The classification of grass family has been traditionally based on morphological and anatomical data. During recent times, the molecular Deoxyribonucleic acid or DNA data have been also used in several studies to understand the phylogenetic relationship within the family (Mathews et al. 2000; Kellogg 2015; Soreng et al. 2015, 2017). According to a recent report, this family can be phylogenic classified into 12 subfamilies, 54 tribes and 109 subtribes, with the Panicoideae subfamily being the largest number in terms of the genera (Soreng et al. 2022).

The genus *Cenchrus* belongs to the subtribe Cenchrinae, tribe Paniceae, subfamily Panicoideae (Soreng et al. 2022). It comprises of about 107 species (including the genus *Pennisetum* s. str.). The genus *Cenchrus* L. and *Pennisetum* Rich. have been traditionally considered to be related genera. Both the genera are differentiated by the characteristics of the bristles subtending the spikelets which are fused well above the base in *Cenchrus*, while those in the *Pennisetum* are free or fused only at the base. Recent molecular phylogenetic studies have confirmed that most species of *Cenchrus* are nested in *Pennisetum*. Several studies strongly suggest to combine the two genera. Thus, with the generic name *Cenchrus* given the priority, all species of *Pennisetum* were transferred to *Cenchrus* (Donadío et al. 2009; Chemisquy et al. 2010; Verloove 2012; Veldkamp 2014).

The genus *Cenchrus* is native to the tropical and subtropical Old World and Americas and introduced to several countries for utilisation (POWO 2023). Several well-known species of this genus are widely planted for forage, such as Napier grass (*C.purpureus* (Schumach.) Morrone), Buffel grass (*C.ciliaris* L.) and Pearl millet (*C.americanus* (L.) Morrone). Some species are cultivated for ornamentation, such as Fountain grass (*C.setaceus* (Forssk.) Morrone), Chinese fountain grass (*C.alopecuroides* (L.) Thunb), Feathertop grass (*C.longisetus* M. C. Johnst.) and White fountain grass (*C.orientalis* (Rich.) Morrone), because of their fluffy inflorescences. However, some species are considered invasive species in several countries, such as Southern sandbur (*C.echinatus* L.), Buffel grass (*C.ciliaris* L.) and Mission grass (*C.setosus* Sw.) (Verloove and Sánchez Gullón 2012; Veldkamp 2014; Global Invasive Species Database 2023).

The usefulness of this genus led to its introduction in Thailand, be it for its ornamental usage or for foraging purposes. Some species are naturalised and considered as invasive and noxious weeds. Even though there have been several studies on grasses in Thailand (Chaisongkram et al. 2013; Teerawatananon et al. 2014; Boonsuk et al. 2016; Wessapak & Ngernsaengsaruay 2021, 2022), the identification of some grasses relies on the literature of neighbouring countries. The species of this genus found in Thailand is yet to be revised. Therefore, the taxonomic treatment presented here will contribute to the “Flora of Thailand” project.

## ﻿Material and methods

The study was based on the herbarium specimens of the following Herbaria: AAU, BK, BKF, BM, C, CMUB, K, KKU, P, PSU and QBG, including digital specimen images from B, BISH, E, G, LINN, S and W. Additional specimens were collected on recent field surveys made throughout Thailand. Plant morphological characters, ecological data and flowering and fruiting periods were also recorded. Most of the grass specimens collected from fieldwork have been submitted to BK and BKF. The identifications were made using morphological characters observed using a stereomicroscope and the available taxonomic literature available in Thailand and the neighbouring region.

## ﻿Taxonomic treatment

### ﻿ Cenchrus

Taxon classificationPlantaePoalesPoaceae

L., Sp. Pl.: 1049. 1753

13BB1AA1-2A33-52C4-BF12-88016B0AFED7

Pennisetum Rich. in Pers., Syn. Pl. 1: 72. 1805. Type species: Pennisetumtyphoideum Rich.
Odontelytrum Hack., Oesterr. Bot. Z. 48: 86. 1898. Type species: Odontelytrumabyssinicum Hack.
Snowdenia C. E. Hubb., Bull. Misc. Inform. Kew 1929: 30. 1929. Type species: Snowdeniamicrocarpha C. E. Hubb.
Kikuyuochloa H. Scholz. Feddes Repert. 117: 513. 2006. Type species: Kikuyuochloaclandestina (Hochst. ex Chiov.) H. Scholz (Basionym: Pennisetumclandestinum Hochst. ex Chiov.)

#### Type species.

*Cenchrusechinatus* L.

#### Description.

Annual or perennial, tufted. ***Culms*** geniculate ascending or prostrate. ***Leaf sheaths*** with or without keeled conspicuous, margins hyaline or hairy. ***Ligules*** ciliolate membrane, ciliolate rim or a fringe of hairs. ***Leaf blades*** linear, margins scabrous, usually chartaceous. ***Inflorescence*** spiciform panicle, usually cylindrical; racemes very short or false spike. ***Spikelets*** 1–8 in cluster, subtended by involucre of bristles and spines, these free at base to forming a cup and burr-like, filiform, terete or flattened, spikelet dorsally compressed; sessile or pedicelled. ***Lower glume*** shorter than spikelet or absent. ***Florets*** 2. ***Lower floret*** male or sterile. ***Lower lemma*** variable. ***Upper floret*** bisexual. ***Lodicules*** present or absent.

A genus of approximately 107 pantropical species, seven species were introduced in Thailand and have become naturalised in the country (the taxonomic treatments and a key to the species excluding the species were introduced so as to be useful in Thailand, but were not found or recorded in their natural habitat).

### ﻿Key to the species

**Table:** 

1	Inner involucre connate into a cup and burr-like forming or connate at least the base (*Cenchrus* s.str.)	**2**
–	Inner involucre free at the base (*Pennisetum* s.str.)	**4**
2	Involucre of a cup and burr-like forming, coriaceous flattened spines connate at lower part as 1–3 mm long; bristles with retrorsely scabrous; upper glume 3 or 5-nerved	**3**
–	Involucre of slightly flattened bristles connate only at the base less than 1 mm long; bristles with antrorsely scabrous; upper glume 1-nerved	**2. *C.ciliaris***
3	Raceme crowded on inflorescence axis; axis internode 0.5–2 mm long	**1. *C.brownii***
–	Raceme loose on inflorescence axis; axis internode 2–3 mm long	**4. *C.echinatus***
4	Culms prostrate, mat-forming; inflorescence very short panicle with central axis less than 1 cm long, concealed within the uppermost leaf sheath; 2–3 racemes along central axis; spikelet 1.6–1.8 cm long; upper glume absent; stigma 2–3 cm long	**3. *C.clandestinus***
–	Culms erect or ascending; inflorescence with central axis more than 1 cm long, exserted from the uppermost leaf sheath; more than 4 racemes along central axis; spikelet less than 1 cm long; upper glume present; stigma less than 1 cm long	**5**
5	Ligules a fringe of hairs; inflorescence axis terete; upper glume shorter than spikelet; lower lemma apex acuminate and chartaceous; upper floret persistent	**6. *C.purpureus***
–	Ligules ciliate membrane; inflorescence axis shortly decurrent ribs; upper glume as long as spikelet; lower lemma apex trifid and membranous; upper floret caducous	**6**
6	Spikelet 2–4 in cluster, one sessile and the other pedicelled spikelets	**5. *C.pedicellatus***
–	Spikelet solitary, sessile spikelet	**7. *C.setosus***

### ﻿1. Cenchrus brownii

Taxon classificationPlantaePoalesPoaceae

Roem. & Schult., Syst. Veg. 2: 258. 1817.

91310ED1-67F9-5CB9-B206-A59E5F6804FB

Figs 1 , 2

≡ Cenchrus inflexus R. Br., Prodr. Fl. Nov. Holland.: 195. 1810.
= Cenchrus viridis Spreng., Syst. Veg. 1: 301. 1824. Type: West Indies, Guadeloupe, C. G. Bertero s.n. (holotype: B [B100248055] seen on digital image). ≡ CenchrusechinatusL.var.viridis (Spreng.) Spreng., Fl. Brit. W. I.: 556. 1864.
= Cenchrus hexaflorus Blanco, Fl. Filip.: 36. 1837. Type: Philippines, Manila, Luzon, Feb 1915, *E. D. Merrill Sp. Blancoan. 811* (neotype, designated by Merrill 1918, pg. 68: US n.v.; isoneotypes: L n.v., P [P00642071!], W n.v.).
= Cenchrus dactylolepis Steud., Syn. Pl. Glumac. 1: 109. 1854. Type: Surinam, 1843, *F. W. R. Hostmann 12a* (holotype: P [P00642090!]; isotype: BAA n.v.).

#### Type.

Australia, Arnheim South Bay, 6 Feb 1803, *R. Brown 6140* (lectotype, designated by DeLisle 1963, pg. 280: BM [BM000795713!]; isolectotypes: E [E00381727] seen on digital image, K [K000215260!], P [P00642070!], US (frag.) n.v.).

#### Description.

Annual, tufted, ***Culms*** geniculate ascending, 30–80 cm high; nodes glabrous; internode subterete, 5–13 cm long, 1–3 mm in diam., glabrous. ***Leaf sheaths*** 4.5–10 cm long, margins membranous, glabrous. ***Ligules*** a fringe of hairs, 0.5–1 mm long. ***Collar*** glabrous. ***Leaf blades*** linear, 14–38 × 0.6–1 cm, apex acute, base rounded, margins scabrous, chartaceous, usually conduplicate, glabrous on both surfaces. ***Inflorescence*** spiciform panicle, 14–43 × 0.8–1.2 cm (including bristles); central axis angular, 5.5–10 cm long, axis internode 0.5–2 mm long, scabrous to glabrescent; peduncle terete, 7–34 cm long, glabrous or scabrous; short racemes crowded along central axis; raceme with (1–)2–3 spikelets in cluster, all sessile, subtended by an involucre of burr-like spines and bristles. ***Involucre*** compose of outer and inner circles; outer circle usually longer than inner circle; outer involucre of bristles, numerous and filamentous, free, 1–6 mm long, one conspicuous longest bristle 4.5–9 mm long, retrorsely scabrous; inner involucre of 6–10 flattened spines, 4–5.2 mm long, connate at lower part 1–3 mm long, burr-like forming, a cup up to 5 mm in diam., coriaceous, puberulose and retrorsely scabrous; involucre falling with spikelets; stipe (raceme-based) 1–2 mm long, hairy. ***Spikelets*** dorsally compressed, lanceolate, 3.6–5.5 × 1–1.7 mm. ***Lower glume*** usually absent, if well-developed, ovate, 0.6–1.2 × 0.3–0.5 mm, apex acute, membranous, glabrous, 1-nerved. ***Upper glume*** ovate or lanceolate-ovate, 3–4 × 1.2–1.8 mm, apex acute or acuminate, membranous to chartaceous, glabrous, 3 or 5-nerved. ***Florets*** 2. ***Lower floret*** male or sterile. ***Lower lemma*** lanceolate, 3.5–5 × 1–1.5 mm, apex acute or acuminate, membranous to chartaceous, glabrous, 3 or 5-nerved. ***Lower palea*** lanceolate-oblong, 3.2–5 × 0.8–1 mm, apex acute or acuminate, margins folded with antrorsely scabrous, membranous, glabrous, 2-nerved or palea absent. ***Upper floret*** bisexual. ***Upper lemma*** lanceolate or ovate-lanceolate, 2.5–5 × 1–1.8 mm, apex acute or acuminate, margins hyaline, coriaceous, glabrous, 5-nerved. ***Upper palea*** lanceolate, 2.5–5 × 0.9–1.5 mm, apex acuminate, margins hyaline, coriaceous, glabrous, 2-keeled, 2-nerved. ***Lodicules*** 2, ca. 0.5 mm long, truncate. ***Stamens*** 3; filament ca. 3 mm long; anther yellow 1–1.6 mm long. ***Pistil*** ovary elliptic, 0.6–1 × 0.2–0.3 mm; style 2; stigma plumose. ***Caryopsis*** obovoid or ellipsoid, gibbous, 1.5–2.5 × 1–1.7 mm.

**Figure 1.**
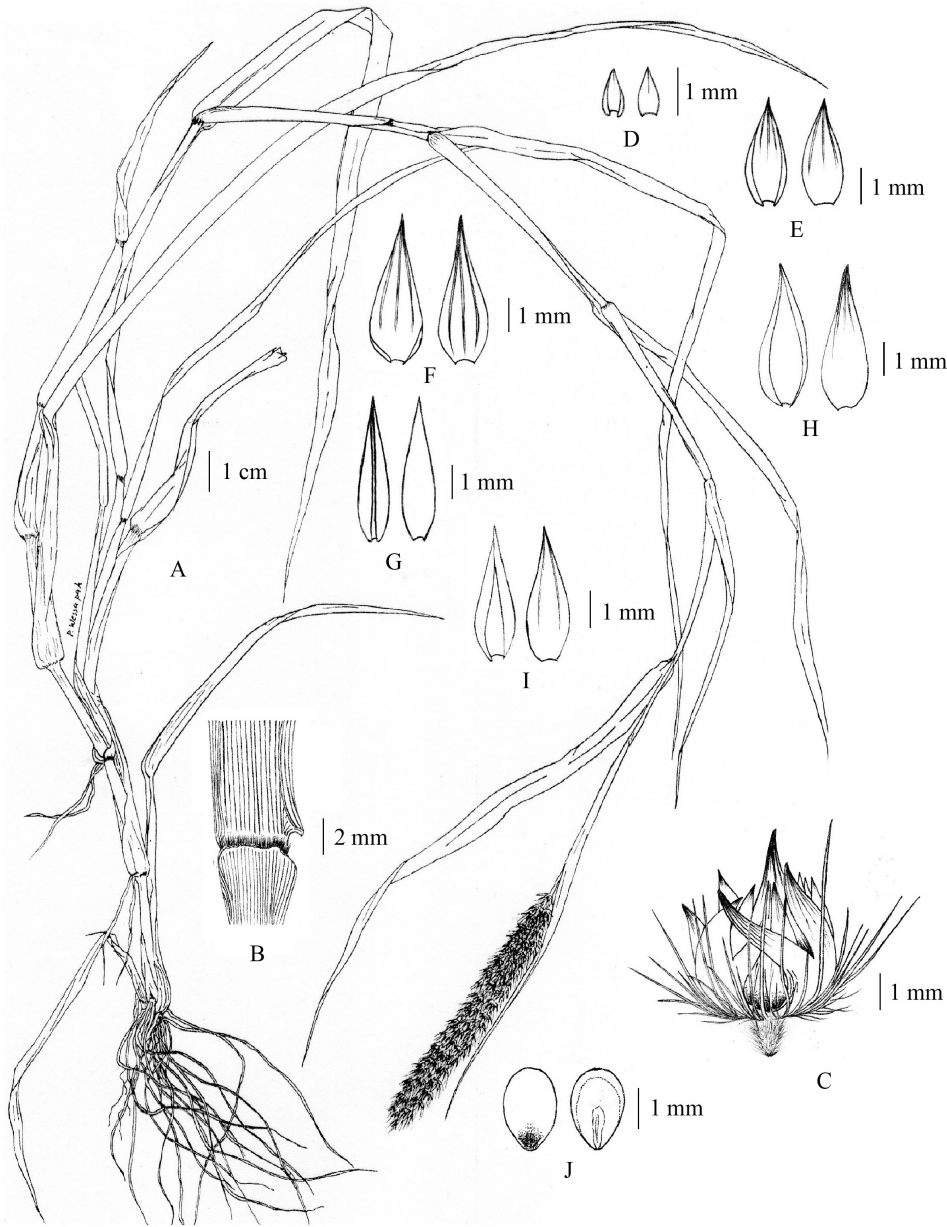
*Cenchrusbrownii* Roem. & Schult **A** habit **B** ligule **C** spikelets with involucre **D** lower glumes **E** upper glumes **F** lower lemmas **G** lower paleas **H** upper lemmas **I** upper paleas **J** caryopsis. (Drawn from *P. Wessapak 365* by Paweena Wessapak).

#### Distribution.

Native to tropical and subtropical America. Introduced to Southeast Asia and Oceania.

#### Distribution in Thailand.

NORTHERN: Chiang Mai (Mae Rim, Doi Suthep-Pui, Mueang Chiang Mai), Tak (Wang Chao Forest, Bhumibol Dam), Sukhothai (Sawan Khalok), Kamphaeng Phet (Phran Kratai), Nakhon Sawan; NORTH-EASTERN: Khon Kaen (Chum Phae); EASTERN: Nakhon Ratchasima (Pak Chong), Amnat Charoen; South-Western: Uthai Thani (Khao Phraya Phai Ruea), Kanchanaburi (Sai Yok, Ban Kao), Ratchaburi (Huai Yang), Prachuap Khiri Khun (Pran Buri, Hat Sai Noi); CENTRAL: Lop Buri (Sap Champa), Suphan Buri (U Thong, Bang Pla Ma), Samut Prakan (Pak Nam), Bangkok (Phu Khao Thong, Chatuchak); SOUTH-EASTERN: Chon Buri (Sattahip, Si Racha), Rayong (Klaeng); PENINSULAR: Chumphon, Ranong (Kraburi), Phuket (Hat Nai Yang), Nakhon Si Thammarat, Satun (Tarutao), Songkhla (Hat Yai).

**Figure 2.**
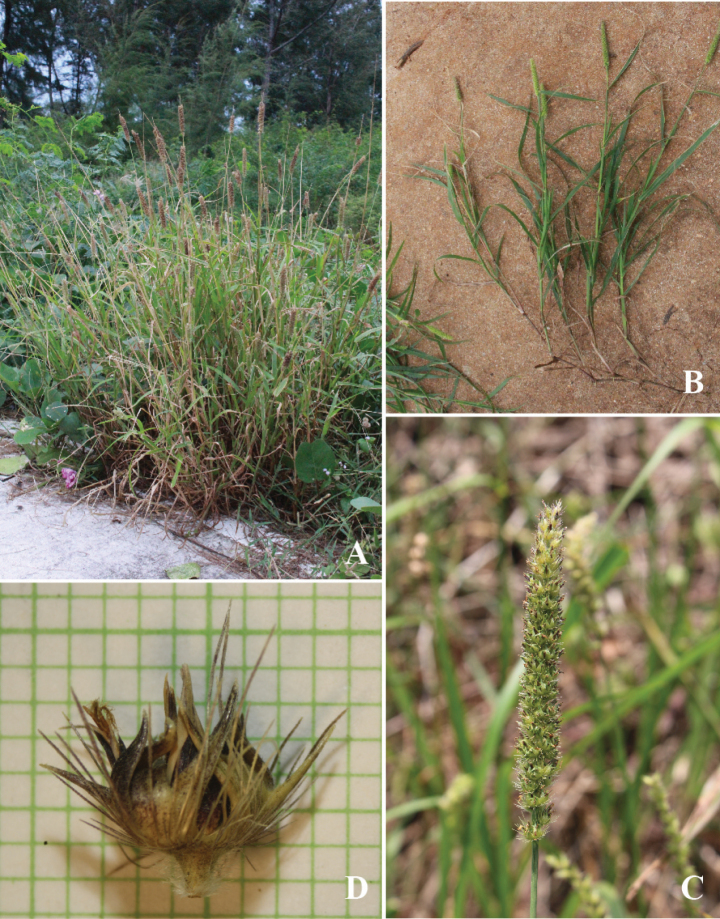
*Cenchrusbrownii* Roem. & Schult **A** habitat **B** habit **C** inflorescence **D** spikelets with involucre. (Photographs: Paweena Wessapak).

#### Habitat and ecology.

In wastelands, open areas by the roadside, the edge of rice fields and the edge of deciduous and evergreen forests at elevations between 0 and 500 m above mean sea level (a.m.s.l.). Flowering and fruiting throughout the year.

#### Vernacular name.

Ya bung (หญ้าบุ้ง); Brown’s burgrass, Brown’s sandbur, Fine-bristle burgrass, Fine-bristle sandbur, Slim-bristle sandbur (English).

#### Specimens examined.

**Thailand. Amnat Charoen**: 28 Oct 2001, *S. Laegaard & M. Norsaengsri 21856* (AAU, BKF, QBG); **Bangkok**: Bang Khen, 12 Nov 1952, *K. Suvatabandhu 33* (BK); Chatuchak, Lat Yao, 26 Dec 2016, *P. Wessapak 346* (BK); Kasetsart University, 12 Sep 2017, *P. Wessapak 393* (BK); Phu Khao Thong, 18 Dec 1955, *T. Smitinand 3147* (BKF); 14 Mar 1920, *A. F. G. Kerr 4063* (BM, K); 5 Jan 1958, *Th. Sørensen, K. Larsen & B. Hansen 22* (C, K); 4 Apr 1958, *Th. Sørensen, K. Larsen & B. Hansen 7904* (BKF, C, K); **Chiang Mai**: Doi Suthep-Pui, 7 Nov 1988, *W. Nanakorn et al. 2296* (QBG); Mae Rim, 8 Aug 1994, *W. Nanakorn et al. 2167* (QBG); ibid., 15 Sep 1995, *W. Nanakorn et al. 4219* (QBG); ibid., 28 Nov 1995, *W. Nanakorn et al. 5377* (QBG); Mueang Chiang Mai, 13 Sep 1995, *W. Nanakorn et al. 4277* (QBG); **Chon Buri**: Sattahip, 26 Nov 1964, *S. Sutheesorn 224* (BK); Si Racha, 8 Nov 1992, *J. F. Maxwell 92-709* (P); 30 May 1964, *C. Hambananda 218* (BKF); **Chumphon**: 6 Apr 1967, *S. Sutheesorn 2127* (BK); **Kamphaeng Phet**: Phran Kratai, Khui Ban Ong, Ban Rai Dong, 22 Oct 1992, *J. F. Maxwell 92-648* (AAU, BKF, P); **Kanchanaburi**: Ban Kao, 8 Nov 1961, *K. Larsen 8044* (C, K); Sai Yok, 15 Aug 2004, *S. Sirimongkol 136* (BKF); Sai Yok, Lum Sum, 17 Feb 2000, *J. F. Maxwell 00-61* (BKF); Sai Yok, Sai Yok Noi, 5 Nov 1979, *T. Shimizu, H. Toyokuni, H. Koyama, T. Yahara & C. Niyomdham T- 21696* (BKF); **Khon Kaen**: Chum Phae, 29 Oct 2001, *S. Laegaard & M. Norsaengsri 21870* (AAU, BKF, QBG); Khon Kaen University, 24 Oct 1982, *P. Chantharanothai 247* (KKU); ibid., 20 Nov 1997, *S. Saensuk s.n.* (KKU); **Lop Buri**: Sap Champa, 19 Nov 1984, *G. Murata, C. Phengklai, S. Mitsuta, T. Yahara, H. Nagamasu & N. Nantasan T-68121* (AAU, BKF); **Nakhon Ratchasima**: Pak Chong, 5 May 1971, *P. Wisuthasom 10* (BK); ibid., 10 Oct 1979, T. *Shimizu, H. Toyokuni, H. Koyama, T. Yahara & T. Santisuk T-18228* (BKF); **Nakhon Sawan**: 21 Jul 1973, *G. Murata, N. Fukuoka & C. Phengklai T-16582* (BKF); **Nakhon Si Thammarat**: 27 May 1995, *W. Nanakorn et al. 3626* (QBG); **Phuket**: Thalaeng, Hat Nai Yang, 12 Sep 1996, *W. Nanakorn et al. 7455* (QBG); **Prachuap Khiri Khun**: Hat Sai Noi, 12 Nov 2017, *P. Wessapak 435* (BK)]; Pran Buri, 11 Nov 2017, *P. Wessapak 430* (BK); 14 Sep 1926, *Put 241* (BK, BM, K); 18 Aug 1967, *T. Shimitzu, N. Fukuoka & A. Nalampoon T-7644* (BKF); **Ranong**: Kraburi, 17 Jan 1987, *J. Supapol 284* (PSU); **Ratchaburi**: Huai Yang, 9 Aug 1966, *K. Larsen, T. Smitinand & E. Warncke 1326* (AAU, C, K, P); **Rayong**: Klaeng, 30 Apr 2017, *P. Wessapak 365* (BK); **Samut Prakan**: Pak Nam, 22 Apr 1924, *A. Marcan 1692* (BM); **Satun**: Tarutao, 23 Oct 1979, *G. Congdon 99* (AAU, PSU); **Songkhla**: Hat Yai, Prince of Songkhla University, 11 Mar 1986, *J. F. Maxwell 86-163* (AAU, PSU); **Sukhothai**: Sawan Khalok, *D. E. Paray 17* (K); **Suphan Buri**: Bang Pla Ma, Phai Kong Din, 17 Sep 2017, *P. Wessapak 399* (BK); U Thong, 26 Mar 2017, *P. Wessapak 355* (BK); **Tak**: Ban Na, 20 May 1959, *T. Smitinand 513* (BK); Rahaeng, 9 Jan 1904, *E. Lindhard 56* (C, K); **Uthai Thani**: Khao Phraya Phai Ruea, 7 Sep 1975, *S. Sutheesorn 3426* (BK).

#### Note.

This species is similar to *Cenchrusechinatus* in terms of having involucre burr-like formation, but differing from the species by having a denser inflorescence, with the involucre usually having a longer outer bristle. In Thailand, it was introduced for foraging and has since become naturalised and is treated as a weed.

### ﻿2. Cenchrus ciliaris

Taxon classificationPlantaePoalesPoaceae

L., Mant. Pl. Altera 2: 302. 1771.

0253DFD0-3535-5548-9DBA-3D85DAD0537C

Figs 3 , 4

≡ Pennisetum ciliare (L.) Link, Hort. Berol. 1: 213. 1827.
= Pennisetum petraeum Steud., Syn. Pl. Glumac. 1: 106. 1854. Type: Iran, Mar 1842, *C. G. T. Kotschy 170* (lectotype, designated by Gutiérrez and Morrone 2012, pg. 264: P [P00642074] seen on digital image; isolectotypes: K [K000244671!], P [P00642073] seen on digital image).
= Cenchrus longifolius Hochst. ex Steud., Syn. Pl. Glumac. 1: 109. 1854. Type: Sudan, Arasch-Cool, 16 Oct 1839, *C. G. T. Kotschy 190* (holotype: P [P00442947!]; isotypes: BM [BM000923378!], E [E00200302] seen on digital image, G n.v., K [K000281252!], P [P00442951!, P00442948!]).

#### Type.

South Africa, Cape, *Koenig s.n*. (lectotype, designated by Clayton & Renvoize in Polhill (ed.) (1982, pg. 691): LINN [LINN-1217.9] seen on digital image).

#### Description.

Perennial, tufted, ***Culms*** geniculate ascending, 45–75 cm high; nodes glabrous; internode subterete, 4–13 mm long, 1–2 mm in diam., glabrous. ***Leaf sheaths*** keeled conspicuous, 4–8 cm long, margins hairy, glabrous or hairy. ***Ligules*** a fringe of hairs, 1–1.5 mm long. ***Collar*** glabrous. ***Leaf blades*** linear, 16–32 cm × 2–7 mm, apex acute, base rounded, margins scabrous, chartaceous, both surfaces hairy or lower surface glabrous and upper surface pilose. ***Inflorescence*** spiciform panicle, 17–33 × 1.5–2.2 cm (including bristles); central axis angular, 6–11 cm long, axis internode 0.5–1.5 mm long, scabrous and puberulose; peduncle terete, 8–25 cm long, scabrous; short raceme along central axis; raceme with (1–)2–3(–4) spikelets in cluster, all sessile, subtended by an involucre of bristles. ***Involucre*** composed of outer and inner circles; outer circle usually shorter than inner circle; outer involucre of bristles, numerous and filamentous, free, 1–7 mm long, antrorsely scabrous; inner involucre of 8–13 slightly flattened bristles with 6–11 mm long, connate only at the base less than 1 mm long, one conspicuous longest bristle 0.9–1.4 cm long, hairy and antrorsely scabrous; involucre falling with spikelets; stipe (raceme-based) ca. 0.5 mm long, hairy. ***Spikelets*** dorsally compressed, lanceolate, 3–4.4 × 1–1.1 mm. ***Lower glume*** ovate, 1.7–2.4 × 0.8–1.2 mm, apex acute, membranous, glabrous, 1-nerved. ***Upper glume*** ovate, 2–3 × 1–1.3 mm, apex acute, membranous, glabrous, 1-nerved. ***Florets*** 2. ***Lower floret*** sterile, rarely male. ***Lower lemma*** lanceolate or ovate-lanceolate, 3–4 × 1–1.5 mm, apex acute or mucronate, membranous, glabrous, 5 or 7-nerved. ***Lower palea*** elliptic or oblong, 2–3 × ca. 0.6 mm, apex obtuse or acute, margins folded with antrorsely scabrous, membranous, glabrous, 2-nerved or palea absent. ***Upper floret*** bisexual. ***Upper lemma*** lanceolate, 3.2–4.3 × 1–1.2 mm, apex acute or acuminate, margins hyaline, chartaceous or coriaceous, glabrous, 5-nerved. ***Upper palea*** lanceolate, 3–4 × 0.7–1.2 mm, apex acuminate, margins hyaline, chartaceous or coriaceous, glabrous 2-keeled, 2-nerved. ***Lodicules*** absent. ***Stamens*** 3; filament ca. 3 mm long; anther yellow 1.5–2.5 mm long. ***Pistil*** ovary elliptic, 0.4–0.8 × 0.2–0.5 mm; style 2; stigma plumose. ***Caryopsis*** ellipsoid, 1.4–1.8 × ca. 1 mm.

**Figure 3.**
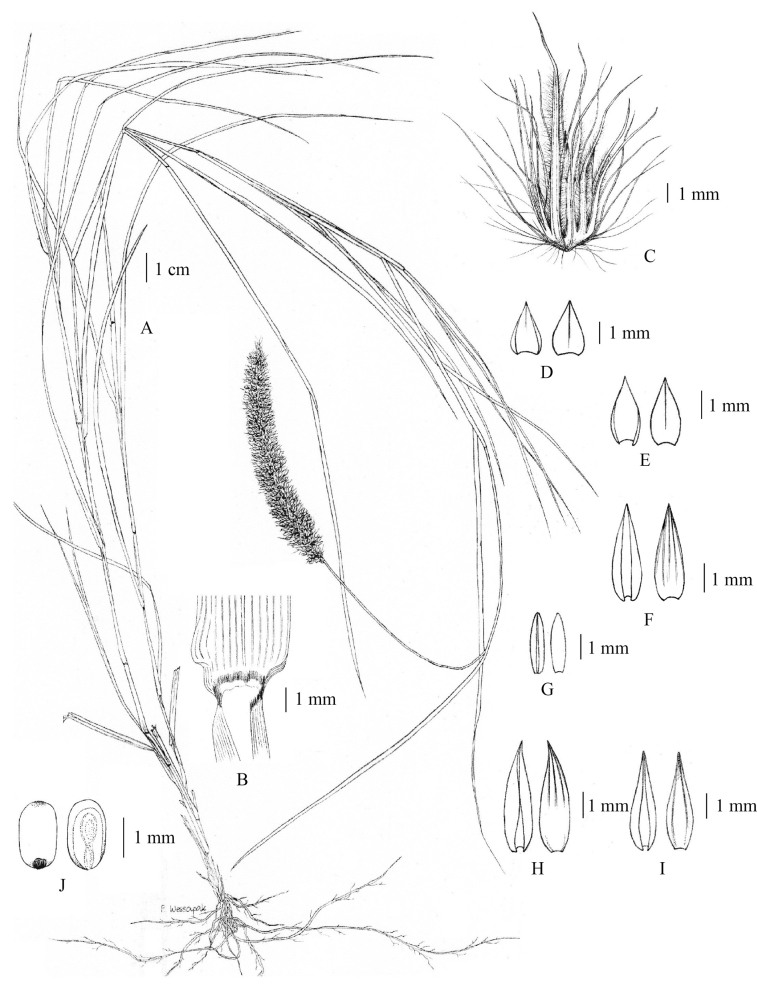
*Cenchrusciliaris* L. **A** habit **B** ligule **C** spikelets with involucre **D** lower glumes **E** upper glumes **F** lower lemmas **G** lower paleas **H** upper lemmas **I** upper paleas **J** caryopsis. Drawn from *P. Wessapak 376* by Paweena Wessapak.

#### Distribution.

Native to Africa, Greece, Sicilia, Middle East, Afghanistan, Pakistan, India and Bangladesh. Introduced to America, Australia and Southeast Asia.

#### Distribution in Thailand.

South-Western: Kanchanaburi (Sai Yok); CENTRAL: Nakhon Pathom (Kamphaengsaen, Mueang Nakhon Pathom).

#### Habitat and ecology.

In open areas by the roadside and the edge of rice fields. This species is cultivated for forage at elevations between 0 and 250 m a.m.s.l. Flowering and fruiting from May to September.

**Figure 4.**
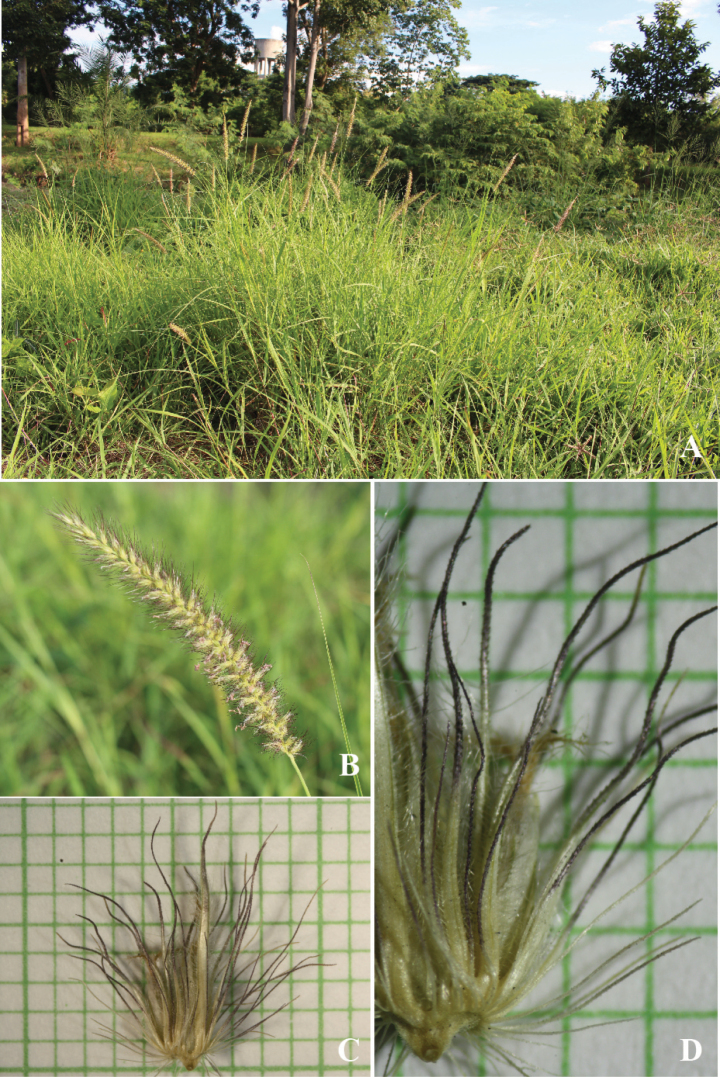
*Cenchrusciliaris* L. **A** habitat **B** inflorescence **C, D** spikelets with involucre. (Photographs: Paweena Wessapak).

#### Vernacular name.

Ya khi khrok rayang phu (หญ้าขี้ครอกรยางค์พู่); African foxtail grass, Buffel grass (English).

#### Specimens examined.

**Thailand. Kanchanaburi**: Sai Yok, 17 May 2005, *P. Porkar 17* (BKF, CMUB); **Nakhon Pathom**: Kamphaengsaen, 22 Jul 2017, *P. Wessapak 373, 374* (BK); Mueang Nakhon Pathom, Thap Luang, 22 Jul 2017, *P. Wessapak 376* (BK).

#### Notes.

This species has been planted as fodder and to prevent soil erosion. It is considered as invasive in some countries. In Thailand, this species commonly escapes planting, but is not widely naturalised like the other species of the *Cenchrus*.

### ﻿3. Cenchrus clandestinus

Taxon classificationPlantaePoalesPoaceae

(Hochst. ex Chiov.) Morrone in Ann. Bot. (Oxford) 106: 127. 2010.

A09E107D-E421-5C5E-9002-BDBF6DEB804E

Fig. 5

Pennisetum clandestinum Hochst. ex Chiov., Annuario Reale Ist. Bot. Roma 8: 41. 1903. Basionym.
≡ Kikuyuochloa clandestina (Hochst. ex Chiov.) H. Scholz in Feddes Repert. 117: 513. 2006.
= Pennisetum inclusum Pilg., Bot. Jahrb. Syst. 45: 209. 1910. Type: Uganda, Lamuru, 3,000 m alt., 30 Jun 1909, *Scheffler 294* (holotype: B [B100168617]; isotypes: BM [BM000923356] seen on digital images, K [K000281293!], P [P00442923] seen on digital image).

#### Type.

Ethiopia, Semien, 27 Oct 1852, *G. H. W. Schimper 2084* (lectotype, designated by Thulin and Phillips (2015, pg. 174): G [G00022569] seen on digital image; isolectotype: S [S-G-4663] seen on digital image).

#### Description.

Perennial, mat-forming, stoloniferous. ***Culms*** prostrate, 5–15 cm high; nodes glabrous; internodes terete or semi-terete, channelled, 1–1.5 cm long, 1–2.5 mm in diam., glabrous. ***Leaf sheaths*** 1.1–1.3 cm long, usually distichous, margins hyaline with hairs, hairy or glabrous on both surfaces. ***Ligules*** a fringe of hairs, 1–2 mm long. ***Collar*** glabrous. ***Leaf blades*** linear, 1.7–7 cm × 2–3.5 mm, apex acute, based rounded or obtuse, chartaceous, usually folded, with or without pilose on both surfaces. ***Inflorescence*** very short spiciform panicle, ca. 2 × 0.2–0.3 cm (including bristles); central axis flattened, 4–5 mm long, scabrous; peduncle very short or sessile; 2–3 short racemes along central axis; raceme with solitary sessile spikelet subtended by an involucre of bristles. ***Involucre*** composed of numerous bristles, slender and filiform, 0.2–1.4 cm long, antrorsely scabrous; involucre falling with spikelet; stipe (raceme-based) absent. ***Spikelets*** dorsally compressed, lanceolate-linear, 1.6–1.8 cm × 1.2–1.8 mm, slightly curved. ***Lower and upper glumes*** absent. ***Florets*** 2. ***Lower floret*** sterile. ***Lower lemma*** lanceolate-linear, ca. 1.5 cm × 1.2–1.8 mm, apex acute, margins folded and membranous, chartaceous, glabrous, 9- or 11-nerved. ***Lower palea*** absent. ***Upper floret*** bisexual, persistent. ***Upper lemma*** lanceolate-linear, 1.6–1.7 cm × 1.2–1.5 mm, apex acute, margins folded and membranous, chartaceous, glabrous, 10- or 11-nerved. ***Upper palea*** lanceolate-linear, 1.4–1.6 cm × 1.2–1.5 mm, apex acute, membranous, glabrous, 4-nerved. ***Lodicules*** absent. ***Stamens*** 3; filament 3.5–5 mm long; anther yellow 2–3.5 mm long. ***Pistil*** ovary lanceolate, 1.8–2 × 0.3–0.4 mm; style 2; stigma plumose, 2–3 cm long, terminally distinct exserted. ***Caryopsis*** not seen.

**Figure 5.**
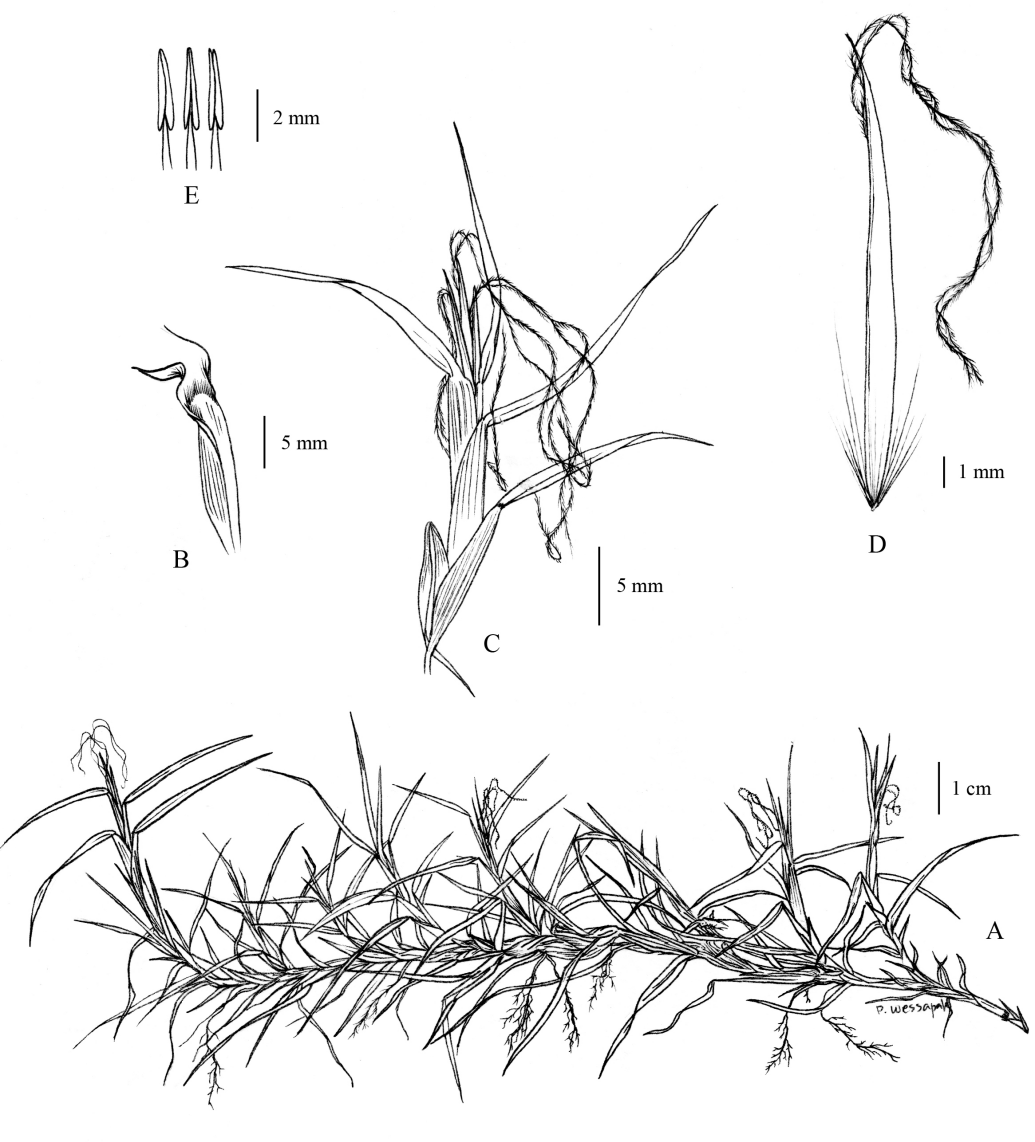
*Cenchrusclandestinus* (Hochst. ex Chiov.) Morrone **A** habit **B** ligule **C** inflorescence enclosed by terminal sheath **D** spikelet with involucre **E** stamens. (Drawn from *S. Laegaard & M. Norsaengsri 21727* by Paweena Wessapak).

#### Distribution.

Native to central-eastern tropical Africa and widely introduced and cultivated elsewhere.

#### Distribution in Thailand.

NORTHERN: Chiang Mai (Doi Inthanon, Mae Taeng).

#### Habitat and ecology.

This species is cultivated as a lawn grass. It grows well on upland and escapes to nearby areas at elevations of 1100–2650 m a.m.s.l. Flowering and fruiting from June to January.

#### Vernacular name.

Kikuyu grass (English).

#### Specimens examined.

**Thailand. Chiang Mai**: Doi Inthanon, 16 Oct 2001, *S. Laegaard & M. Norsaengsri 21727* (AAU); Mae Taeng, Huai Thung Cho, 13 Jan 1986, *Y. Paisooksantivathana y1760-86* (BK); Mae Taeng, Pa Pae, Thung Cho, 3 Jun 1981, *Y. Paisooksantivathana & T. Chuaycharoern y616-81* (BK).

#### Note.

This species is unique to the *Cenchrus* as it has a short spiciform inflorescence, partially exserted from the uppermost leaf sheath. Originally, this species was native to the tropical parts of Africa and has been widely introduced elsewhere for forage, urban landscaping, preventing soil erosion and is also considered as a weed in some countries (Hosaka 1958; Cudney et al. 1993; Arango-Gaviria et al. 2019). In Thailand, there are a few specimens recorded in the natural habitat, maybe because its inflorescence is difficult to notice and its vegetative part is similar to other lawn grasses.

### ﻿4. Cenchrus echinatus

Taxon classificationPlantaePoalesPoaceae

L., Sp. Pl.: 1050. 1753.

904F188F-77A1-5336-B008-61F518365C21

Figs 6 , 7

= Cenchrus pungens Kunth, Nov. Gen. Sp. 1: 115. 1816. Type: Peru, Guyaquil, F. W. H. A. von Humboldt & A. J. A. Bonpland s.n. (holotype: P [P00669360] seen on digital image).
= Cenchrus brevisetus E. Fourn., Mexic. Pl. 2: 50. 1886. Type: Mexico, Orizaba, 1866, *E. Bourgeau 3140* (lectotype, designated by Gutiérrez and Morrone 2012, pg. 266: G [G00099346], seen on digital image; isolectotypes: BR n.v., K [K000643125!]). ≡ CenchrusechinatusL.var.brevisetus (E. Fourn.) Scribn., Publ. Field Columb. Mus., Bot. Ser. 2: 26. 1900.
= Cenchrus insularis Scribn. ex Millsp., Publ. Field Columb. Mus., Bot. Ser. 2: 26. 1896. Type: Mexico, Yucatan, Pagaros Island, 8 Mar 1899, *C. F. Millspaugh 1759* (holotype: F n.v.; isotypes: B [B100278941] seen on digital image, US n.v.).
= Cenchrus hillebrandianus Hitchc., Mem. Bernice Pauahi Bishop Mus., Honolulu 8: 211. 1922. Type: Hawaii, Oahu, Waikiki, 19 Jun 1916, *A. S. Hitchcock 13801* (holotype: US n.v.; isotypes: B [B100278942], BISH [BISH-439425] seen on digital images, K [K001056124!], P [P00698357] seen on digital image). ≡ CenchrusechinatusL.var.hillebrandianus (Hitchc.) F. Br., Bull. Bernice P. Bishop Mus. 84: 65. 1931.
= Cenchrus echinatus L. var. glabratus F. Br., Bull. Bernice P. Bishop Mus. 84: 66. 1931. Type: Marquesas Islands, Nuku Hiva, *F. Brown 745* (lectotype, designated by St. John 1976, pg. 419: BISH [BISH-92743] seen on digital image).
= Cenchrus echinatus L. var. pennisetoides F. Br., Bull. Bernice P. Bishop Mus. 84: 66. 1931. Type: Marquesas Islands, Nuku Hiva, Hakaui, 15 Jul 1921, *F. B. H. Brown & E. D. W. Brown 657* (holotype: BISH [BISH-92748] seen on digital image).

#### Type.

Jamaica, ‘Habitat in Jamaica, Curassa’, *Anonymous s.n.* (lectotype, designated by Veldkamp in Jarvis et al. (ed.) (1993, pg. 31): L [Herb. A. van Royen No. 912.356-116] n.v.; isolectotype: L [Herb. A. van Royen No. 912.356-103] n.v.).

#### Description.

Annual, tufted, ***Culms*** geniculate ascending, 15–75 cm high; nodes glabrous; internode subterete, 5–15 cm long, 1–2 mm in diam., glabrous. ***Leaf sheaths*** keeled conspicuous, 5–9 cm long, margins hyaline, glabrous. ***Ligules*** a fringe of hairs, 0.8–1.2 mm long. ***Collar*** glabrous. ***Leaf blades*** linear, 14–32 cm × 4–8 mm, apex acute, base rounded, margins scabrous, chartaceous, glabrous on both surfaces, sometimes upper surface pilose. ***Inflorescence*** spiciform panicle, 16–35 × 0.9–1.6 cm (including bristles); central axis angular, 5–11 cm long; axis internode 2–3 mm long, scabrous to subglabrous; peduncle terete, 10–27 cm long, glabrous or scabrous; short racemes loose along central axis; raceme with 2–4(–6) spikelets in cluster, all sessile, subtended by involucre of burr-like spines and bristles. ***Involucre*** composed of outer and inner circles; outer circle usually shorter than inner circle; outer involucre of bristles, numerous and filamentous, free, 1–4(–5) mm long, retrorsely scabrous; inner involucre of 7–11 flattened spines, 4–7 mm long, connate at lower part as 1–3 mm long, burr-like forming, a cup up to 5 mm in diam., coriaceous, puberulose and retrorsely scabrous; involucre falling with spikelets; stipe (raceme-based) 0.5–1.3 mm long, hairy, falling with raceme. ***Spikelets*** dorsally compressed, lanceolate, 4.5–6 × 1–2 mm. ***Lower glume*** ovate or lanceolate, 1–3 × 0.3–1.6 mm, apex acute or obtuse, membranous, glabrous, 1-nerved or nerveless, rarely absent. ***Upper glume*** lanceolate, 3.2–5.5 × 1.2–1.8 mm, apex acute or obtuse, membranous to chartaceous, glabrous, 3 or 5-nerved. ***Florets*** 2. ***Lower floret*** male or sterile. ***Lower lemma*** lanceolate, 3.8–5.8 × 1–2 mm, apex acute or acuminate, membranous to chartaceous, glabrous, 5-nerved. ***Lower palea*** lanceolate-oblong, 3.5–5.5 × 0.5–1 mm, apex acute to acuminate, margins folded with retrorsely scabrous, membranous, glabrous, 2-nerved, sometimes palea absent. ***Upper floret*** bisexual. ***Upper lemma*** lanceolate, 3.3–6 × 1–2 mm, apex acute or acuminate, margins hyaline, coriaceous, glabrous, 3 or 5-nerved. ***Upper palea*** lanceolate, 3–5.7 × 1–2 mm, apex acute or acuminate, margins hyaline, coriaceous, glabrous, 2-keeled, 2-nerved. ***Lodicules*** absent. ***Stamens*** 3; filament ca. 3 mm long; anther yellow, 1–2 mm long. ***Pistil*** ovary elliptic, 0.4–0.8 × 0.2–0.3 mm; style 2; stigma plumose. ***Caryopsis*** ellipsoid and gibbous or obovoid and slightly flattened, 1.5–3 × 1–2 mm.

**Figure 6.**
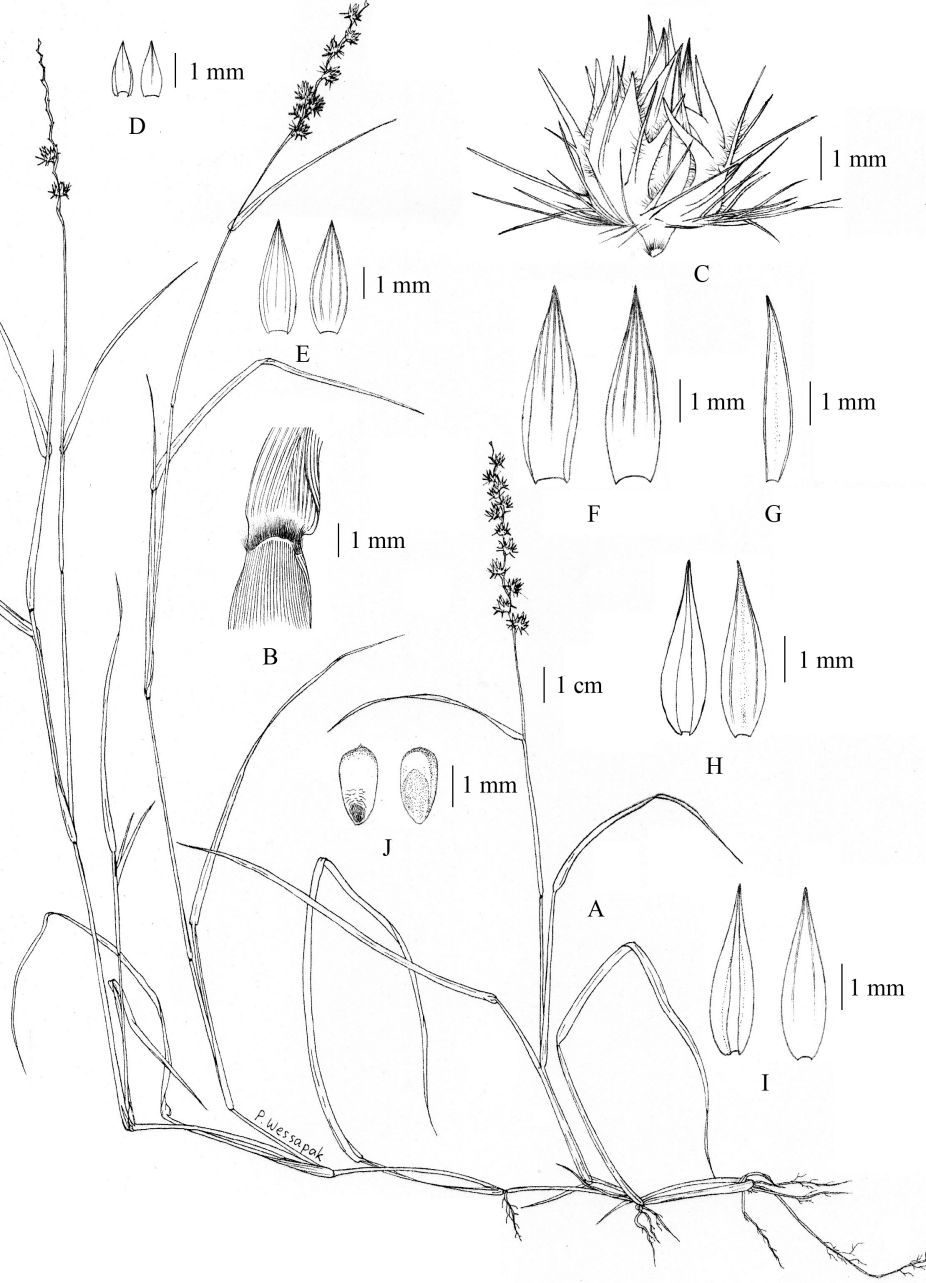
*Cenchrusechinatus* L. **A** habit **B** ligule **C** spikelets with involucre **D** lower glumes **E** upper glumes **F** lower lemmas **G** lower paleas **H** upper lemmas **I** upper paleas **J** caryopsis. (Drawn from *P. Wessapak 361* by Paweena Wessapak).

#### Distribution.

Native to North America and South America. Introduced and naturalising elsewhere in tropical and temperate zones worldwide.

#### Distribution in Thailand.

NORTHERN: Tak (Thararak Waterfall); NORTH-EASTERN: Loei (Phu Ruea, Na Haeo), Udon Thani (Ban Dung), Khon Kaen (Mueang Khon Kaen, Phu Wiang, Khok Pho Chai); EASTERN: Nakhon Ratchasima; SOUTH-WESTERN: Kanchanaburi (Thong Pha Phum), Prachuap Khiri Khan (Pran Buri); CENTRAL: Saraburi (Muak Lek), Bangkok (Chatuchak); SOUTH-EASTERN: Chon Buri (Samaesan, Phanat Nikhom), Rayong (Klaeng); PENINSULAR: Songkhla (Mueang Songkhla, Hat Yai).

**Figure 7.**
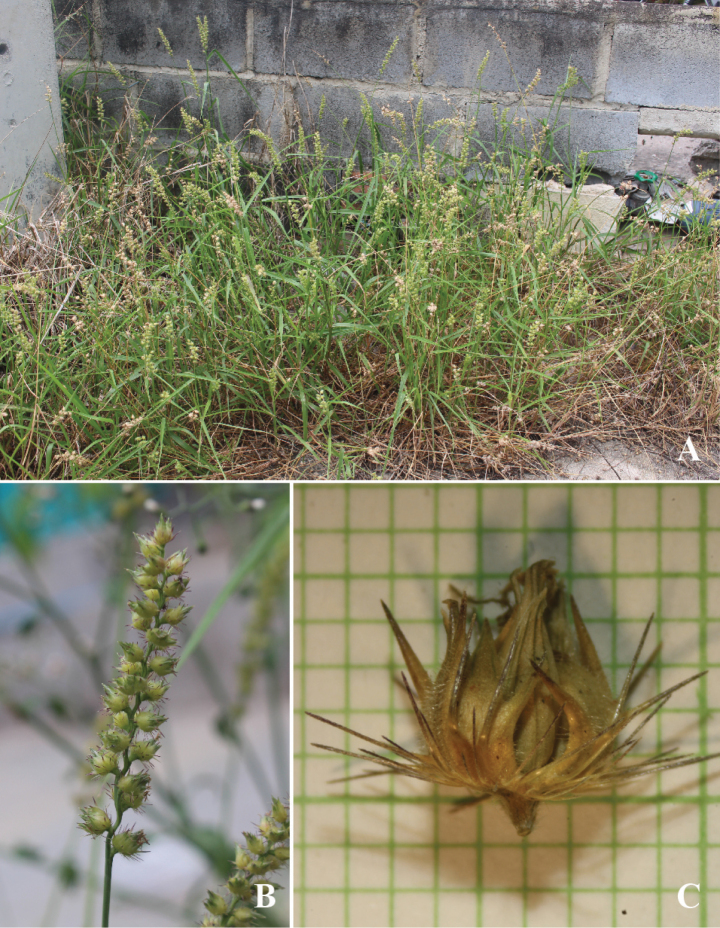
*Cenchrusechinatus* L. **A** habitat **B** inflorescence **C** spikelets with involucre. (Photographs: Paweena Wessapak).

#### Habitat and ecology.

In wastelands, open areas by the roadside, the edge of rice fields and the edge of deciduous and evergreen forests at elevations between 0 and 600 m a.m.s.l. Flowering and fruiting throughout the year.

#### Vernacular name.

Ya khi khrok (หญ้าขี้ครอก), **Ya son krachap** (หญ้าสอนกระจับ); Hedgehog grass, Mossman river grass, Southern sandbur, Spiny sandbur (English).

#### Specimens examined.

**Thailand. Bangkok**: Chatuchak, Kasetsart University, 25 Dec 2016, *P. Wessapak 347* (BK); ibid., 12 Sep 2017, *P. Wessapak 394* (BK); 2 Mar 1987, *J. Lambinon 87/10* (AAU)]; **Chon Buri**: Phanat Nikhom, Kut Ngong, 29 Apr 2017, *P. Wessapak 361* (BK); Samaesan, 8 Aug 1998, *T. Wongprasert s.n.* (BKF); **Kanchanaburi**: Thong Pha Phum, Pilog, Ban Pilog, 29 Jun 2004, *S. Sirimongkol 123* (BKF); **Khon Kaen**: Khok Pho Chai, 24 Feb 2010, *P. Thongson 14* (QBG); Mueang Khon Kaen, 25 Oct 2001, *S. Laegaard & M. Norsaengsri 21791* (AAU, BKF, K, SING, QBG); Phu Wiang, 15 Sep 1994, *W. Nanakorn et al. 1730* (QBG); **Loei**: Na Haeo, 20 Jun 1995, *W. Nanakorn et al. 3829* (QBG); Phu Ruea, 24 Jul 2004, *C. Jaroenchai 76* (KKU); **Nakhon Ratchasima**: 4 Sep 1989, *Pasikarn & Prayad 41* (BK); **Prachuap Khiri Khan**: Pran Buri, 11 Nov 2017, *P. Wessapak 431* (BK); **Rayong**: Klaeng, 30 Apr 2017, *P. Wessapak 364* (BK); **Saraburi**: Muak Lek, Sap Sanun, 9 Jul 2017, *P. Wessapak 372* (BK); **Songkhla**: Hat Yai, 23 Nov 2016, *P. Wessapak, C. Ngernsaengsaruay, N. Meeprom & W. Boonthasak 337* (BK); Mueang Songkhla, 6 Oct 1990, *B. Aksorn 6* (PSU); Sadao, 30 Jul 2013, *H. Soh 24* (PSU); **Tak**: Mae Sot, Thararak Waterfall, 22 Jun 2005, *R. Pooma, K. Phattarahirankanok, S. Sirimongkol & M. Poophat 5426* (BKF); ibid., 21 Aug 2010, *M. Norsaengsri 7094* (QBG); **Udon Thani**: Ban Dung, Ban Dung Yai, 4 Dec 2008, *M. Norsaengsri 4550* (QBG).

#### Note.

The species is considered as a noxious weed in some countries (Ensbey 2014; Verloove and Sánchez Gullón 2012). In Thailand, it was introduced for forage and has become naturalised and treated as a weed.

### ﻿5. Cenchrus pedicellatus

Taxon classificationPlantaePoalesPoaceae

(Trin.) Morrone in Chemisquy et al., Ann. Bot. (Oxford) 106: 128. 2010.

CBAAD709-D483-55CB-B922-324A84C35B65

Figs 8 , 9

Pennisetum pedicellatum Trin., Mém. Acad. Imp. Sci. Saint-Pétersbourg, Sér. 6, Sci. Math., Seconde Pt. Sci. Nat. 3(2): 184. 1834. Basionym.
= Pennisetum lanuginosum Hochst., Flora 27: 252. 1844. Type: Sudan, Oct 1839, *C. G. T. Kotschy 394* (lectotype, designated by Gutiérrez 2015, pg. 164: TUB [TUB018061-cb006519] n.v.; isolectotypes: BM [BM000923370!], K [K000281241!], W [W0000305, W18890244992] seen on digital images).
= Pennisetum amoenum Hochst. ex A. Rich., Tent. Fl. Abyss. 2: 386. 1850. Type: Ethiopia, Oct 1839, *G. H. W. Schimper 641* (lectotype, designated by Gutiérrez and Morrone 2012, pg. 267: P [P03471044!]; isolectotypes: B [B100167868] seen on digital image, BM [BM000923371!], W [W18890244973] seen on digital image).

#### Type.

Cape Verde Islands, St. Iaga, *D. Peters s.n.* (holotype: LE [Herb. Trinius 1102.1] n.v.).

#### Description.

Annual, tufted. ***Culms*** erect or ascending, 0.5–2 m high; nodes glabrous; internodes subterete, sometimes slightly flattened, 5.5–30 cm long, 1.5–9 mm in diam., glabrous. ***Leaf sheaths*** 7–13 mm long, margins with hairs or tubercle-base hairs, glabrous. ***Ligules*** ciliate membrane, 2–4 mm long. ***Collar*** glabrous. ***Leaf blades*** linear, 20–50 × 0.5–1.7 cm, apex acute, base rounded with tubercle-base hairs, margins scabrous, chartaceous, hairy on both surfaces. ***Inflorescence*** spiciform panicle, 22–35 × 2.5–3 cm (including bristles); central axis with shortly decurrent ribs, 11–19 cm long, scabrous or pubescent; peduncle terete, 10–20 cm long, villose or scabrous; short racemes along central axis; raceme with 2–4 spikelets in cluster, one sessile and the other pedicelled, sessile spikelet smaller than pedicelled spikelet; pedicels 1–3 mm long, hairy; subtended by the involucre bristles. ***Involucre*** composed of outer and inner circles, numerous and free at base, outer circle shorter than inner circle; outer involucre 2–5 mm long; inner involucre 1–2 cm long, one conspicuous longest bristle 1.6–2.5 cm long, antrorsely scabrous and woolly hairs; involucre falling with spikelets; stipe (raceme-based) absent. ***Spikelets*** dorsally compressed, lanceolate, 3.5–5 × 0.8–1.4 mm. ***Lower glume*** lanceolate or lanceolate-elliptic, 1–2.5 × 0.5–1 mm, apex bifid, hyaline, pubescent, nerveless or 1-nerved. ***Upper glume*** lanceolate or elliptic, 3.5–5 × 1–1.3 mm, apex acuminate, cuspidate or mucronate, membranous, glabrous or scabrous, 5-nerved. ***Florets*** 2. ***Lower floret*** male. ***Lower lemma*** lanceolate, 3–4 × 0.8–1.2 mm, apex trifid, membranous, glabrous or scabrous, 5-nerved. ***Lower palea*** lanceolate or oblong-lanceolate, 3–4 × 0.4–0.6 mm, apex acute to acuminate, margins folded, membranous, glabrous, 2 or 3-nerved. ***Upper floret*** bisexual, caducous. ***Upper lemma*** lanceolate, 1.8–2.8 × 0.5–1 mm, apex obtuse with ciliate, coriaceous, glabrous, 3-obscure or 5-nerved. ***Upper palea*** lanceolate, 1.8–2.8 × 0.5–1 mm, apex obtuse with ciliate, coriaceous, glabrous, nerveless. ***Lodicules*** absent. ***Stamens*** 3; filament 1.5–2 mm long; anther yellow, 1.5–2.2 mm long. ***Pistil*** ovary oblong or elliptic, 0.4–0.8 × 0.1–0.2 mm; style 2; stigma plumose, 2.8–4 mm long. ***Caryopsis*** ellipsoid, 1.3–2 × 0.6–1 mm.

**Figure 8.**
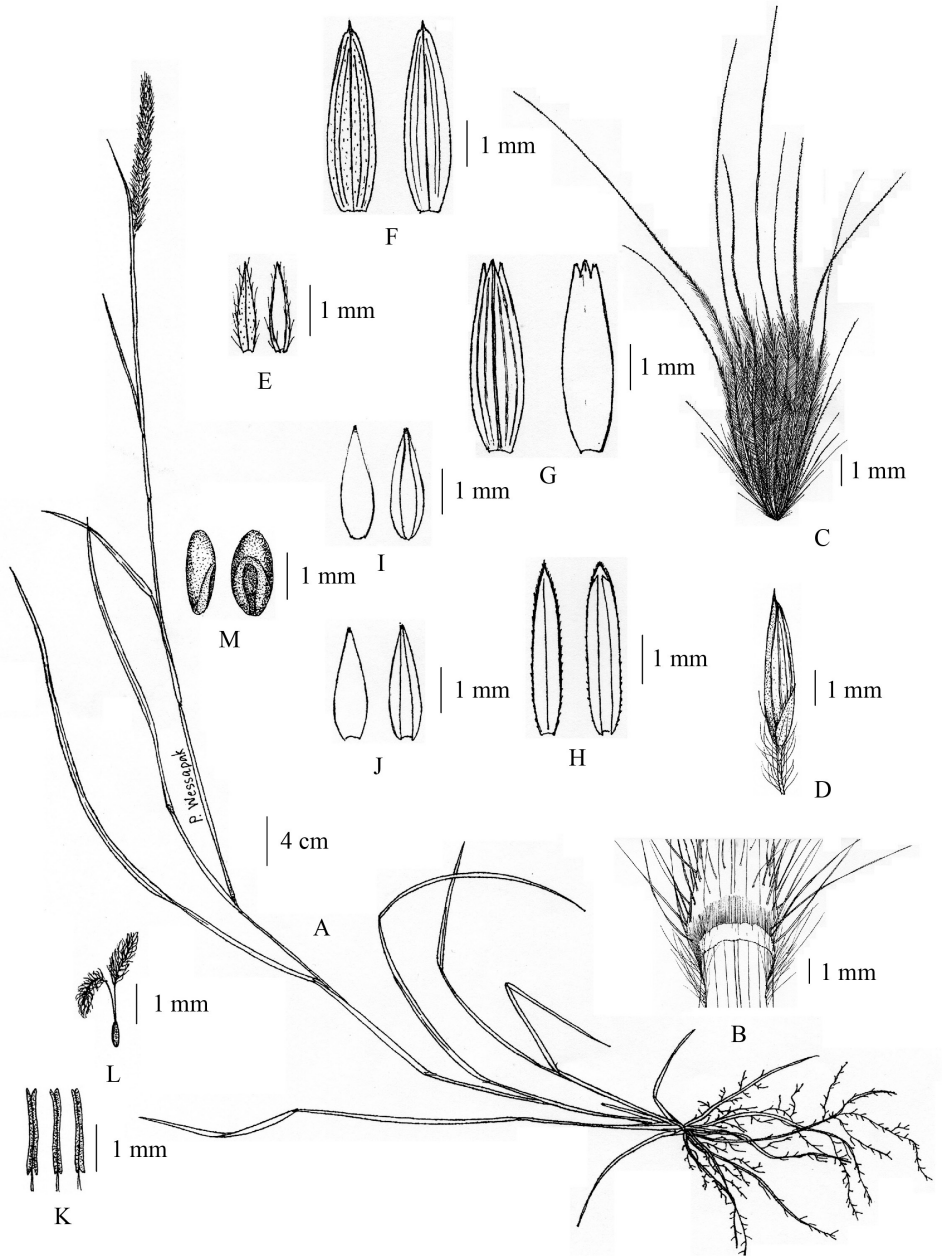
*Cenchruspedicellatus* (Trin.) Morrone **A** habit **B** ligule **C** spikelet with involucre **D** pedicelled spikelet **E** lower glumes **F** upper glumes **G** lower lemmas **H** lower paleas **I** upper lemmas **J** upper paleas **K** stamens **L** pistil **M** caryopsis. (Drawn by Paweena Wessapak).

#### Distribution.

Originally form Tropical Africa, India and Macronesia and introduced elsewhere.

#### Distribution in Thailand.

NORTHERN: Chiang Mai (Mae Rim, Doi Suthep-Pui, Doi Saket, Mae Chaem, Samoeng, Mueang Chiang Mai), Lamphun (Doi Khun Tan), Tak; NORTH-EASTERN: Loei (Phu Luang, Phu Ruea, Phu Kradueng), Udon Thani (Ban Dung, Phu Kao), Nong Khai, Maha Sarakham, Khon Kaen (Phu Wiang, Tha Phra); EASTERN: Nakhon Ratchasima (Pak Thong Chai, Pak Chong), Buri Ram (Chaloem Phra Kiat), Si Sa Ket (Khao Phra Wihan), Ubon Ratchathani (Pha Taem, Phu Chong Na Yoi); SOUTH-WESTERN: Kanchanaburi (Thong Pha Phum); CENTRAL: Bangkok, Suphan Buri (Mueang Suphan Buri), Nakhon Nayok (Khao Yai), Samut Prakan (Phra Pradaeng); SOUTH-EASTERN: Prachin Buri (Khao Khiao), Chon Buri (Ko Si Chang, Sattahip), Sakaeo (Aranyaprathet).

**Figure 9.**
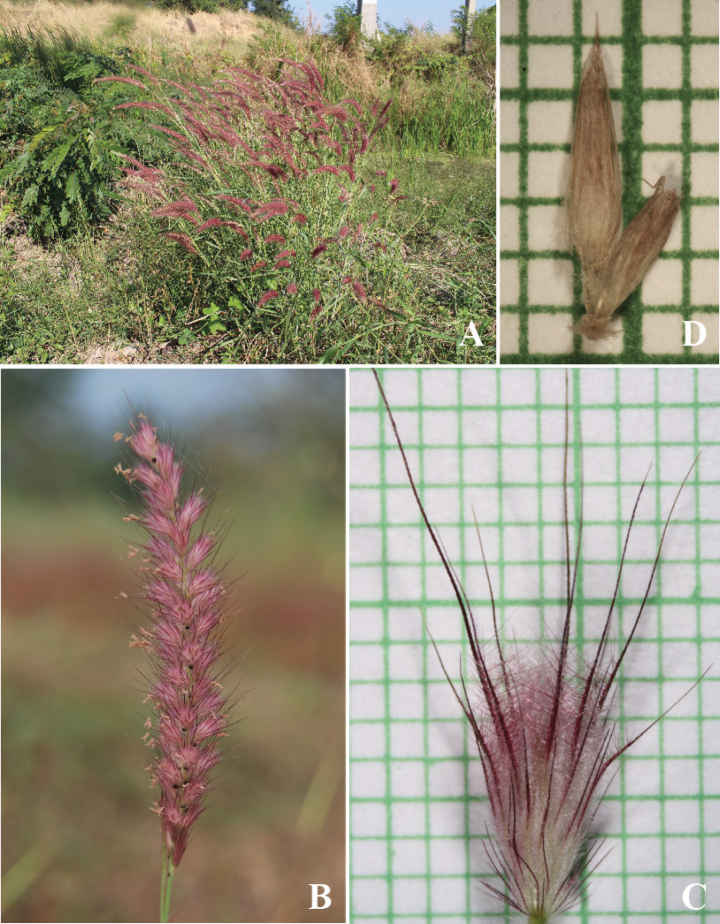
*Cenchruspedicellatus* (Trin.) Morrone **A** habitat **B** inflorescence **C** spikelets with involucre **D** spikelets. (Photographs: Paweena Wessapak).

#### Habitat and ecology.

In open areas by the roadside, open areas in disturbed or deciduous forest at elevations of 25–1100 m a.m.s.l. Flowering and fruiting from October to March.

#### Vernacular name.

Ya kha chon chop (หญ้าขจรจบ), **Ya kha chon chop dok yai** (หญ้าขจรจบดอกใหญ่); Annual kyasuwa grass, Deenanath grass, Dinanath grass, Hairy fountain grass (English).

#### Specimens examined.

**Thailand. Bangkok**: 16 Dec 1990, *K. Larsen, S. S. Larsen, W. Nanakorn, W. Ueachirakan & P. Sirirugsa 42014* (AAU); **Buri Ram**: Chaloem Phra Kiat, Isan Khet, 21 Oct 2017, *P. Wessapak 409* (BK); **Chiang Mai**: Doi Saket, Ban Pang Faen, 10 Dec 1998, *F. Konta & S. Khao-Iam 4422* (BKF); Doi Suthep, 21 Oct 1987, *J. F. Maxwell 87-1218* (AAU, BKF); Doi Suthep-Pui, 21 Nov 1996, *S. K. Kafle 13* (CMUB); Mae Chaem, Ban Mae Wak, 19 Dec 1998, *F. Konta, C. Phengklai & S. Khao-Iam 4813* (BKF); Mae Rim, 10 Feb 2006, *C. Glamwaewwong 466* (QBG); Mueang Chiang Mai, 5 Dec 1989, *C. Lek-korbkul 602* (CMUB); Samoeng, 21 Oct 2001, *S. Laegaard 21763* (AAU, BKF, K, QBG); **Chon Buri**: Ko Si Chang, 29 Nov 1992, *J. F. Maxwell 92-785* (CMUB, P); Sattahip, Thung Brong, 10 Jun 1971, *J. F. Maxwell 71-416* (AAU, BK); **Kanchanaburi**: Thong Pha Phum, Pilok, 23 Oct 2004, *S. Sirimongkol 166* (BKF); **Khon Kaen**: Phu Wiang, Khok Phu Ta Ka, 30 Nov 2003, *C. Jaroenchai 36* (KKU); Tha Phra, 3 Nov 1971, *T. Smitinand 11518* (BKF); **Lamphun**: Mae Tha, Doi Khun Tan, 18 Nov 1993, *J. F. Maxwell 93-1376* (BKF, CMUB); **Loei**: Phu Kradueng, 25 Dec 2011, *M. Norsaengsri & N. Tathana 8424* (QBG); Phu Luang, 19 Dec 2001, *V. Chaemchumroon 1204* (BKF); Phu Ruea, 27 Nov 2005, *C. Jaroenchai 243* (KKU); **Maha Sarakham**: 30 Oct 1965, *S. Sutheesorn 705* (BK); **Nakhon Nayok**: Khao Yai, 13 Oct 1984, *G. Murata, C. Phengklai, S. Mitsuta, H. Nagamasu & N. Nantasan T-52211* (BKF); ibid., 15 Oct 1984, *G. Murata, C. Phengklai, S. Mitsuta, H. Nagamasu & N. Nantasen T-52591* (BKF); **Nakhon Ratchasima**: Pak Chong, 14 Jan 1965, *Umpai 172* (BK); Pak Thong Chai, Nov 1970, *Ch. Charoenpol, K. Larsen & E. Warncke 4535* (BKF, K); 9 Dec 1962, *C. Phengklai 408* (BKF); 6 Nov 1963, *T. Smitinand 8398* (BKF); **Nong Khai**: 10 Nov 1984, *G. Murata, S. Mitsuta, T. Yahara, H. Nagamasu & N. Nantasen T-40452* (BKF); **Prachin Buri**: Mueang Prachin Buri, Khao Khiao, 25 Oct 2000, *S. Watthana & T. Riyapun 963* (QBG); **Sakaeo**: Aranyaprathet, 20 Nov 1984, *Chabeuf s.n.* (P); **Samut Prakan**: Phra Pradaeng, Song Khanong, 25 Mar 2012, *P. Wessapak & C. Ngernsaengsaruay 205* (BK); **Si Sa Ket**: Khao Phra Wihan, Pha Mo I Daeng, 21 Dec 2005, *R. Pooma, K. Pattarahirankanok, S. Sirimongkol & K. Poopath 6002* (BKF); 10 Oct 1984, *G. Murata, C. Phengklai, S. Mitsuta, H. Nagamasu & N. Nantasen T-38118* (BKF)]; **Suphan Buri**: Mueang Suphan Buri, Don Masang, 30 Dec 2016, *P. Wessapak 352* (BK)]; **Tak**: Mae Sot-Umphang road, 23 Oct 1997, *Anonymous s.n.* (QBG); **Ubon Ratchathani**: Pha Taem, 28 Oct 2001, *S. Laegaard, M. Norsaengsri, P. Pornpongrungrueng & S. Khoomkrathok 21857* (AAU, QBG); Phu Chong Na Yoi, Phalan Kong Kwian, 6 Nov 2010, *P. Wessapak, Y. Buangam & W. Sareemongkonnimit 158* (BK); **Udon Thani**: Ban Dung, Ban Dung Yai, 4 Dec 2008, *M. Norsaengsri 4561* (QBG); Phu Kao, Non Sung, 3 Jan 1968, *K. Bunchuai 1519* (BKF, C).

#### Note.

This species has been used for improving pasture quality, in soil remediation and erosion control (Asmare et al. 2016; Umer et al. 2019; Kumar and Fulekar 2022). It was introduced to Thailand from India for forage. At present, it has become naturalised as a weed and is distributed to almost all Thai floristic regions. *Cenchruspedicellatus* is a close relative of *C.setosus*, but differs in terms of having a pedicelled spikelet, while *C.setosus* only has one sessile spikelet. Furthermore, its hairs on bristles are usually fluffier than the latter.

### ﻿6. Cenchrus purpureus

Taxon classificationPlantaePoalesPoaceae

(Schumach.) Morrone in Chemisquy et al., Ann. Bot. (Oxford) 106: 129. 2010.

AF0B7630-45F7-5832-9A2F-A4C8B2221946

Figs 10 , 11

Pennisetum purpureum Schumach., Beskr. Guin. Pl.: 44. 1827. Basionym.
= Pennisetum macrostachyum Benth. in W. J. Hooker, Niger Fl.: 563. 1849, non (Brongn.) Trin. (1834). Type: Nigeria, Aboh, Vogel s.n. (lectotype, designated here: K [K000281312!]; isolectotype: K [K000281311!]).
= Pennisetum flavicomum Leeke, Z. Naturwiss. 79: 46. 1907. Type: Tanzania, *von Prittwitz & Gaffron 199* (holotype: B [B100167864] seen on digital image).
= Pennisetum pruinosum Leeke, Z. Naturwiss. 79: 46. 1907. Type: Tanzania, Mtemere am Rufidji, *W. Goetze 66* (holotype: B [B100167822] seen on digital image).
= Pennisetum pallescens Leeke, Z. Naturwiss. 79: 47. 1907. Type: Togo, Nov 1902, *O. Kersting 719* (holotype: B [B100167821] seen on digital image).

#### Type.

Ghana, *P. Thonning 355* (lectotype, designated by Hepper (1976, pg. 149): C [C10004308!]; isolectotype BM n.v.).

#### Description.

Perennial, tufted. ***Culms*** erect or ascending, 1.8–4 m high; nodes glabrous or hairy; internodes terete or subterete, 7–32 cm long, 2–10 mm in diam., glabrous. ***Leaf sheaths*** 9–20 cm long, glabrous. ***Ligules*** a fringe of hairs, 2–5 mm long. ***Collar*** glabrous. ***Leaf blades*** linear, 26–100 × 0.4–3(–3.5) cm, apex acute, base rounded glabrous or with tubercle-base hairs, margins scabrous, chartaceous, scabrous on both surfaces (sometimes hairy on upper surface). ***Inflorescence*** spiciform panicle, 45–80 × 2–4 cm (including bristles); central axis terete, 15–40 cm long, pubescent; peduncle terete, 25–55 cm long, glabrous or pubescent; short racemes along central axis; raceme with 1–4 spikelets in cluster, one sessile and the other pedicelled (if solitary, it is sessile or subsessile spikelet), pedicelled spikelets smaller than sessile spikelet; pedicels 0.5–2.2 mm long, scabrous; subtended by involucre bristles. ***Involucre*** composed of outer and inner circles, numerous and free at base, outer circle shorter than inner circle; outer involucre 2–4 mm long; inner involucre 1–1.4 cm long (sometimes up to 2.6 cm long), one conspicuous longest bristle 1.1–2.3 cm long, antrorsely scabrous; involucre falling with spikelets; stipe (raceme-based) 0.2–0.3 mm long, hairy, persistent on axis. ***Spikelets*** dorsally compressed, lanceolate, 4–6.5 × 0.8–1.3 mm. ***Lower glume*** ovate or lanceolate, 0.5–1.5 × 0.2–0.6 mm, apex acute or acuminate, sometimes obtuse, chartaceous, glabrous, nerveless or 2-obscure-nerved. ***Upper glume*** lanceolate or ovate-lanceolate, 1.8–4.2 × 0.4–1 mm, apex acute or acuminate, chartaceous, glabrous with or without scabrous along nerve, 1- or 3-nerved or nerveless. ***Lower floret*** male or sterile. ***Lower lemma*** lanceolate, 3–6 × 0.8–1.3 mm, apex acuminate, chartaceous, glabrous with or without scabrous along nerve, 3- or 5-nerved. ***Lower palea*** mostly absent or lanceolate, 4.5–5.2 × 0.8–1 mm, apex acute, margins fold with or without scabrous, membranous, glabrous, 2- or 3-nerved. ***Upper floret*** bisexual, persistent. ***Upper lemma*** lanceolate, 4–6.5 × 0.8–1.2 mm, apex acuminate, margins with ciliate, coriaceous, glabrous and scabrous near tip, 3- or 5-nerved. ***Upper palea*** lanceolate, 4.3–6.5 × 0.8–1.2 mm, apex acuminate, coriaceous, glabrous with or without scabrous near tip, 2-, 3- or 5-nerved. ***Lodicules*** absent. ***Stamens*** 3, filament 0.3–3 mm long, anther yellow, 1.5–2.8 mm long. ***Pistil*** ovary oblong-lanceolate, 0.6–1.5 × 0.1–0.5 mm; style 2, 1.5–4 mm long; stigma plumose, 2–6 mm long. ***Caryopsis*** ellipsoid, 1.5–2 × 0.8–1 mm.

**Figure 10.**
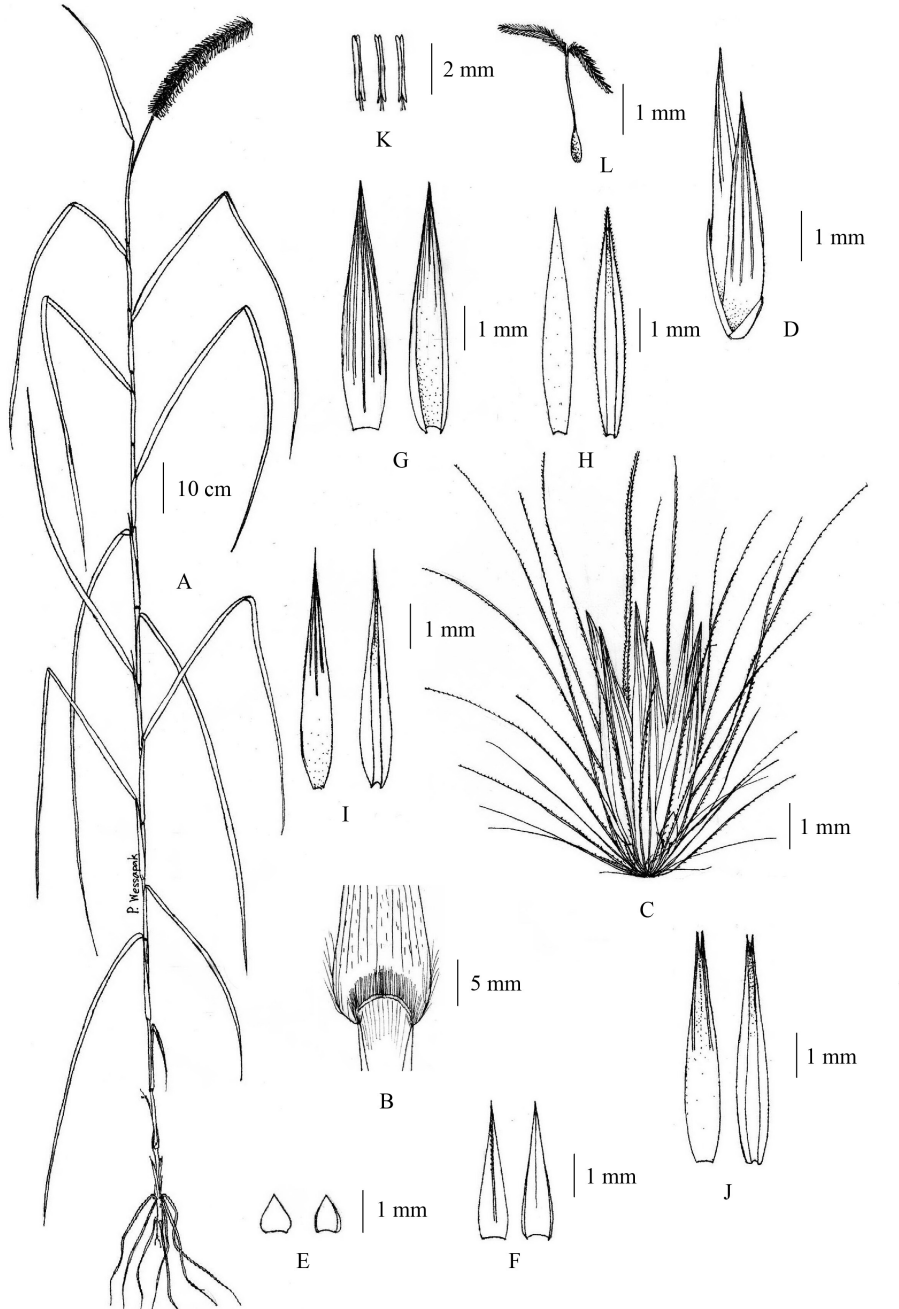
*Cenchruspurpureus* (Schumach.) Morrone **A** habit **B** ligule **C** spikelets with involucre **D** spikelet **E** lower glumes **F** upper glumes **G** lower lemmas **H** lower paleas **I** upper lemmas **J** upper paleas **K** stamens **L** pistil. (Drawn by Paweena Wessapak).

#### Distribution.

Native from Sahara to Tropical Africa and introduced to China, India, Myanmar, Indo-China, Malaysia, Australia, New Zealand, North America and South America.

#### Distribution in Thailand.

NORTHERN: Mae Hong Son (Pang Ma Pha, Mae Rim, Doi Suthep-Pui), Chiang Rai (Doi Tung, Mae Sai, Tham Luang-Khun Nam Nang Non), Lampang (Ngao); NORTH-EASTERN: Khon Kaen; EASTERN: Nakhon Ratchasima (Pak Chong), Si Sa Ket (Phrai Bueng); CENTRAL: Nakhon Pathom (Kampheangsaen); SOUTH-EASTERN: Chon Buri (Sattahip), Rayong (Klaeng); PENINSULAR: Surat Thani (Tha Chang), Krabi (Khlong Thom), Trang (Khao Chong), Songkhla (Sadao), Yala (Bannang Sata), Narathiwat (Sungei Kolok).

**Figure 11.**
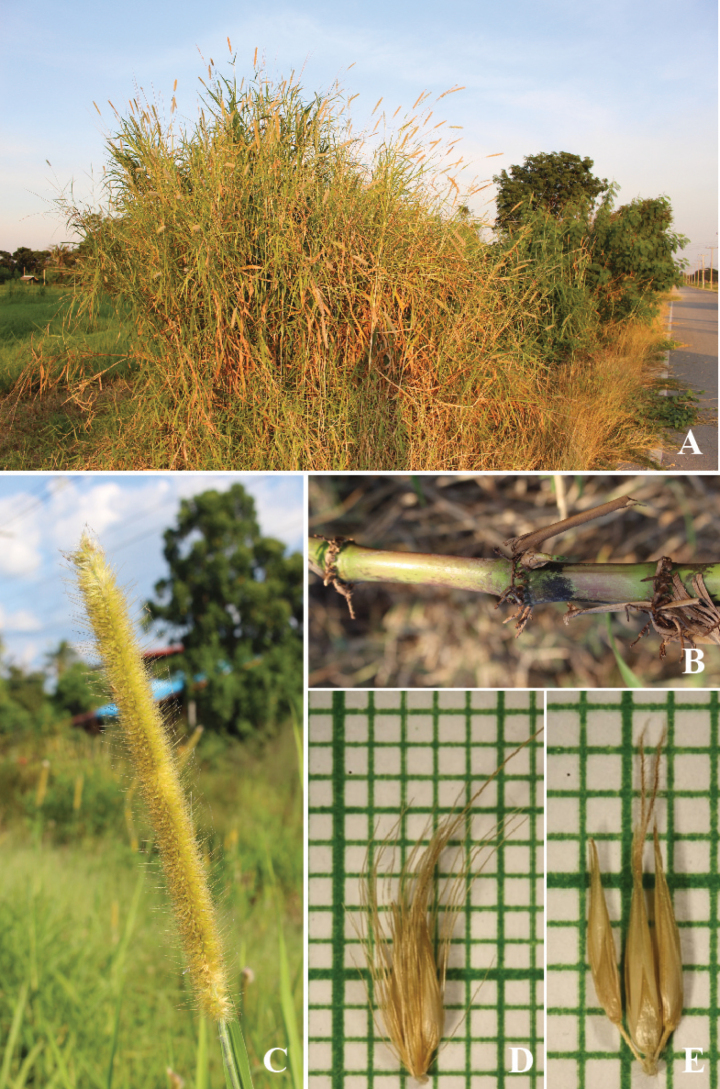
*Cenchruspurpureus* (Schumach.) Morrone **A** habitat **B** culm with node rooting **C** inflorescence **D** spikelets with involucre **E** spikelets. (Photographs: Paweena Wessapak).

#### Habitat and ecology.

In open areas by the roadside, disturbed sites and the edge of deciduous forests. They are also cultivated for forage at elevations of 50–1350 m a.m.s.l. Flowering and fruiting from October to March.

#### Vernacular name.

Ya hang chang (หญ้าหางช้าง), **Ya Napier** (หญ้าเนเปียร์); Elephant grass, Napier grass (English).

#### Specimens examined.

**Thailand. Chiang Mai**: Doi Suthep-Pui, 23 Jan 1991, *J. F. Maxwell 91-95* (AAU); ibid., 1 Jan 2001, *J. F. Maxwell* 01-1 (BKF, CMUB); Doi Suthep, 4 Nov 1958, *Th. Sorensen, K. Larsen & B. Hansen 2633* (BKF, C, K)]; ibid., 24 Dec 1965, *E. Hennipman 3478* (BKF, C); ibid., 19 Mar 1997, *K. Chayamarit & C. Pattanacharoen 732* (BKF); Mae Rim, 28 Nov 1995, *Anonymous 5378* (QBG); Mae Rim, Queen Sirikit Botanic Garden, 10 Feb 2006, *C. Glamwaewwong 463* (QBG); **Chiang Rai**: Doi Tung, Mae Fa Luang, 23 Dec 2006, *J. F. Maxwell 06-989* (QBG, CMUB); Mae Sai, Pong Pha, Ban Nam Cham, 2 Dec 2008, *M. Norsaengsri 4475* (QBG); Tham Luang-Khun Nam Nang Non, 13 Feb 2012, *M. Norsaengsri & N. Thatana 8876* (QBG); **Chon Buri**: Sattahip, 11 Oct 1969, *J. F. Maxwell s.n.* (BK)]; **Khon Kaen**: Khon Kaen University, 25 Dec 1997, *U. Pattaramanon 18* (KKU); **Krabi**: Khlong Thom, Khlong Thom Tai, 12 Nov 2011, *P. Wessapak 202* (BK); **Lampang**: Ngao, Ban Huat, 9 Dec 2014, *J. F. Maxwell 14-19* (CMUB, PSU); **Mae Hong Son**: Pang Ma Pha, Ban Tha Khrai, 16 Feb 2005, *K. Pruesapan KP2005-9* (BK); **Nakhon Pathom**: Kampheangsaen, 28 Dec 2016, *P. Wessapak 348*, *350* (BK); **Nakhon Ratchasima**: Pak Chong, Oct 1931, *A. F. G. Kerr s.n.* (BM, BK); **Narathiwat**: Sungei Kolok, Nikhom Waeng, 6 Mar 1974, *K. Larsen & S. S. Larsen 33030* (AAU, BKF, K); **Rayong**: Klaeng, Chak Pong, Ban Phlong Sawai, 13 Jan 2009, *P. Wessumritt & M. Norsaengsri 160* (QBG); **Si Sa Ket**: Phrai Bueng, Suk Sawat, 26 Dec 2004, *A. Virapongse 194* (BKF); **Songkhla**: Sadao, Samnak Taeo, 24 Oct 2010, *P. Wessapak & C. Ngernsaengsaruay 153* (BK, BKF); 11 Nov 1959, *T. Smitinand 6124* (BKF); **Surat Thani** Tha Chang, Tha Koei, Ban Thong Sai, 2 Nov 2011, *P. Wessapak 200* (BK); **Trang**: Khao Chong, 18 Nov 1990, *K. Larsen, S. S. Larsen, M. S. Barfod, W. Nanakorn, W. Ueachirakan & P. Sirirugsa 41606* (AAU, BKF, PSU); **Yala**: Bannang Sata, 12 Dec 1961, *Ploenchit 1775* (BKF).

#### Notes.

Napier grass is a multipurpose forage crop. It is mainly used to feed livestock and is also used for ornamentation and soil erosion control. Its fibres are used to make pulp and in papermaking. Furthermore, its biofuel producing potential is being researched because all the parts of this species produce lignocellulose biomass which can be used to produce cheap biofuel (Obi Reddy et al. 2014; Chavre and Sonawane 2021). This species was introduced to Thailand from Malaysia for forage. At present, it has become naturalised to almost all Thai floristic regions.

**Figure 12.**
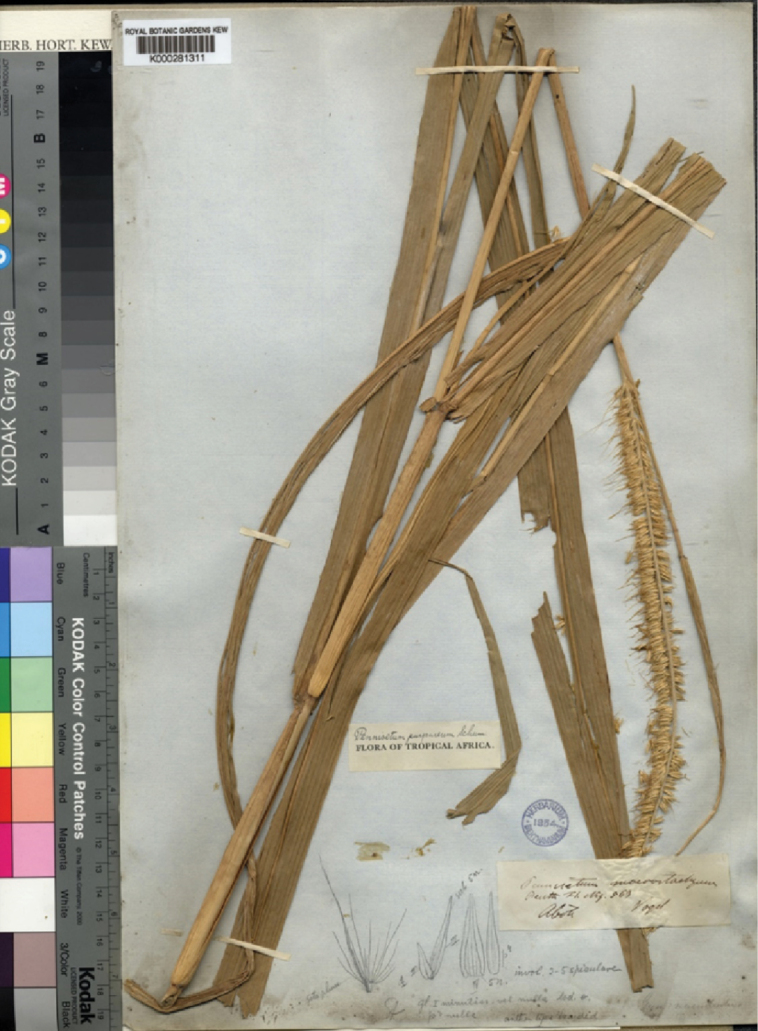
Lectotype of *Pennisetummacrostachyum* Benth. Digital image The Board of Trustees of the Herbarium of the Royal Kew Botanic Garden (K).

The synonym, *Pennisetummacrostachyum*, was described by George Bentham in 1849 and is based on the Vogel’s specimens from Aboh (Nigeria) and Fernando Po (Equatorial Guinea). He did not choose any specimens to be the holotype. We located the Vogel’s specimen in three sheets at K which was collected from Aboh (Nigeria) [K000281311 and K000281312] and Fernando Po (Equatorial Guinea) [K000281310] and, according to Art 9.6 of the ICN (Turland et al. 2018), they constitute syntypes. The sheet K000281312 is a well-preserved specimen with more leaves and spikelets and, therefore, it is herein designated as the lectotype for the synonym *P.macrostachyum*. (Fig. 12)

### ﻿7. Cenchrus setosus

Taxon classificationPlantaePoalesPoaceae

Sw., Prodr.: 26. 1788.

AEFDD2CB-DD89-581D-924F-0918ABEA3AEF

Figs 13 , 14

≡ Pennisetum setosum (Sw.) Rich, Syn. Pl. 1: 72. 1805.
≡ Pennisetum polystachion (L.) Schult. subsp. setosum (Sw.) Brunken, Bot. J. Linn. Soc. 79(1): 63. 1979.

#### Type.

Jamaica, *O. Swartz s.n.* (lectotype, designated by Hitchcock (1908, pg. 143): S [S-R-969] seen on digital image; isolectotypes: BM [BM000938799!], G [G00168094], S [S-R-968, S06-637] seen on digital images).

#### Description.

Annual or perennial, tufted. ***Culms*** erect or ascending, 0.5–3 m high; nodes glabrous; internodes subterete, sometimes slightly flattened, 5.5–34 cm long, 1–8 mm in diam., glabrous. ***Leaf sheaths*** 0.7–2.5 cm long, glabrous. ***Ligules*** ciliate membrane, 1.5–3 mm long. ***Collar*** ciliate or hairs 1–2 mm long. ***Leaf blades*** linear, 10–85 × 0.4–2.2 cm, apex acute, base rounded with tubercle-base hairs, margins scabrous, chartaceous, hairy on both surfaces. ***Inflorescence*** spiciform panicle, 22–55 × 1.5–4.5 cm (including bristles); central axis with shortly decurrent ribs, 12–23 cm long, scabrous or almost glabrous; peduncle terete, 10–35 cm long, scabrous or almost glabrous or glabrous; short racemes along central axis; raceme with solitary sessile spikelet subtended by an involucre bristle. ***Involucre*** composed of outer and inner circles, numerous and free at base, outer circle shorter than inner circle; outer involucre 1–5 mm long; inner involucre 1–1.4 cm long, one conspicuous longest bristle 1.2–3.3 cm long, antrorsely scabrous and hairy. Involucre falling with spikelet; stipe (raceme-based) absent. ***Spikelets*** dorsally compressed, lanceolate, 3.3–4.5 × 0.8–1.2 mm. ***Lower glume*** absent, minute ca. 0.1 mm long or triangular, 0.5–0.8 × 0.1–0.6 mm, apex acute, membranous, nerveless. ***Upper glume*** lanceolate, 3.3–4.5 × 0.8–1.2 mm, apex acuminate or mucronate, membranous, glabrous or slightly scabrous, 5-nerved. ***Florets*** 2. ***Lower floret*** male or sterile. ***Lower lemma*** lanceolate-elliptic or lanceolate, 2.8–3.5 × 0.8–1.2 mm, apex trifid, membranous, glabrous or slightly scabrous, 3- or 5-nerved. ***Lower palea*** oblong or oblong-lanceolate, 2.2–3.2 × 0.3–0.8 mm, apex acute, margins folded, membranous, glabrous, 2 or 3-nerved or nerveless, sometime palea absent. ***Upper floret*** bisexual, caducous. ***Upper lemma*** lanceolate, 1.9–2.5 × 0.4–0.8 mm, apex obtuse with ciliate, coriaceous, glabrous, nerveless or 5-obscure-nerved. ***Upper palea*** lanceolate, 1.9–2.5 × 0.3–0.8 mm, apex obtuse with ciliate, coriaceous, glabrous, nerveless or 5-obscure-nerved. ***Lodicules*** absent. ***Stamens*** 3; filament 1–1.5 mm long; anther yellow or brown 1.3–2.2 mm long. ***Pistil*** ovary oblong or elliptic, 0.3–0.6 × 0.1–0.2 mm; style 2, 1–2 mm long; stigma plumose, 2–3 mm long. ***Caryopsis*** ellipsoid, 1.2–1.7 × 0.5–0.8 mm.

**Figure 13.**
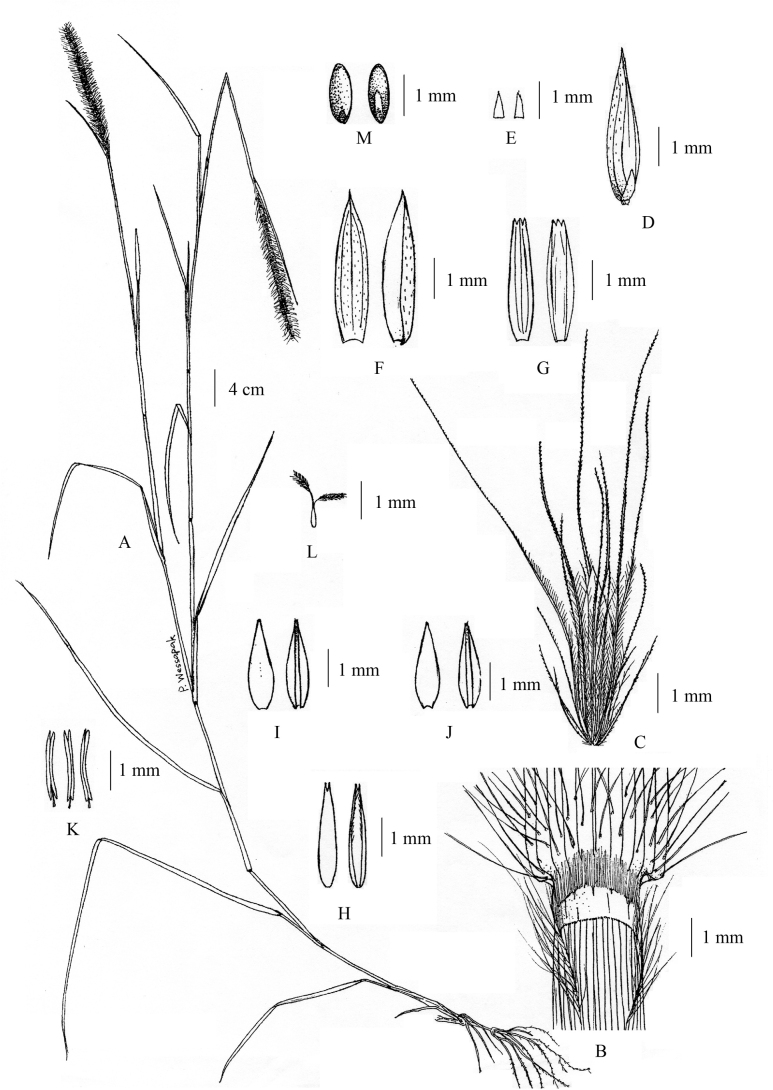
*Cenchrussetosus* Sw. **A** habit **B** ligule **C** spikelet with involucre **D** spikelet **E** lower glumes **F** upper glumes **G** lower lemmas **H** lower paleas **I** upper lemmas **J** upper paleas **K** stamens **L** pistil **M** caryopsis. (Drawn by Paweena Wessapak).

#### Distribution.

Native to India and Tropical Africa and introduced to Indo-China, Malaysia, Australia, North America and South America.

#### Distribution in Thailand.

NORTHERN: Chiang Mai (Doi Saket, Mae Ya Waterfall, Chom Thong, Mae Rim, Doi Chiang Dao, Hang Dong), Chiang Rai (Mae Chan, Mae Sai, Mueang Chiang Rai), Phrae (Song), Nan (Tham Sa Koen), Lampang (Doi Luang), Phitsanulok (Thung Salaeng Luang), Kamphaeng Phet (Pang Sila Thong); NORTH-EASTERN: Phetchabun (Nam Nao), Loei (Phu Ruea, Wang Saphung), Udon Thani (Kumphawapi), Khon Kaen (Waeng Yai, Phu Wiang, Nam Nao); EASTERN: Nakhon Ratchasima (Pak Thong Chai, Sakaerat, Pak Chong, Khao Yai), Buri Ram (Chaloem Phra Kiat), Ubon Ratchathani (Phu Chong Na Yoi); SOUTH-WESTERN: Kanchanaburi (Thong Pha Phum); CENTRAL: Saraburi (Wang Muang), Nakhon Pathom (Phutthamonthon), Nakhon Nayok (Khao Yai), Samut Prakan (Phra Pradaeng); SOUTH-EASTERN: Prachin Buri (Kabin Buri), Chon Buri (Si Racha, Ban Bueng), Rayong (Klaeng), Chanthaburi (Tha Mai, Khlong Nong Khla, Laem Sadet), Trat (Ko Chang); PENINSULAR: Surat Thani (Chaiya), Phuket (Thalaeng), Krabi (Ao Ma Ya, Khlong Thom), Nakhon Si Thammarat (Thung Song, Tha Yang), Phatthalung (Phanang Tung), Trang (Sikao), Satun (Mueang Satun), Songkhla (Hat Yai, Khao Phra, Rattaphum, Sadao, Na Mhom).

**Figure 14.**
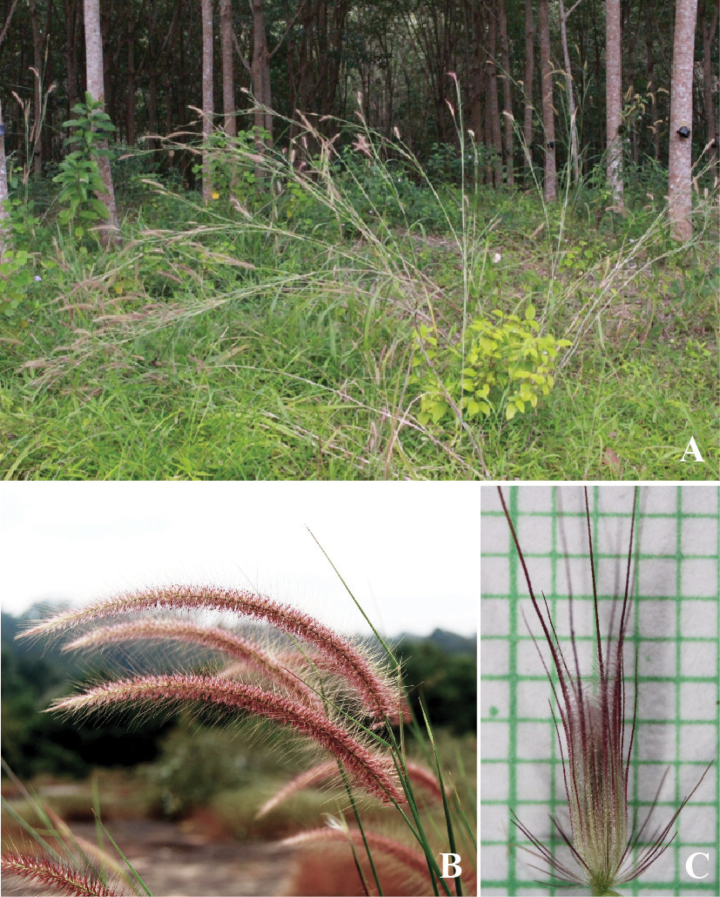
*Cenchrussetosus* Sw. **A** habitat **B** inflorescences **C** spikelet with involucre. (Photographs: Paweena Wessapak).

#### Habitat and ecology.

In open areas by the roadside, disturbed and open areas in deciduous forests at elevations between 0 and 1200 m a.m.s.l. Flowering and fruiting throughout the year.

#### Vernacular name.

Ya kha chon chop (หญ้าขจรจบ), **Ya kha chon chop dok lek** (หญ้าขจรจบดอกเล็ก), Ya kha chon chop dok lueang (หญ้าขจรจบดอกเหลือง); Mission grass (English).

#### Specimens examined.

**Thailand. Buri Ram**: Chaloem Phra Kiat, Isan Khet, 21 Oct 2017, *P. Wessapak 410* (BK); **Chanthaburi**: Tha Mai, 2 Jan 1971, *S. Sutheesorn 1947* (BK); ibid., 8 Nov 1971, *S. Sutheesorn 2017* (BK); Tha Mai, Khlong Nong Khla, 16 Feb 2009, *M. Norsaengsri 4894* (QBG); Tha Mai, Laem Sadet, 24 Feb 2008, *M. Norsaengsri & K. Wangwasit 3487* (BKF, QBG); **Chiang Mai**: Chom Thong, 3 Dec 1991, *J. F. Maxwell 91-1092* (AAU); Doi Chiang Dao, 21 Nov 1999, *P. Suksathan 2221* (QBG); Hang Dong, 21 Mar 2009, *S. Watthana 3007* (QBG); Doi Saket, Ban Pang Faen, 10 Dec 1998, *F. Konta & S. Khao-Iam 4421* (BKF); Mae Rim, 3 Oct 1994, *W. Nanakorn et al. s.n.* (QBG); Mae Ya Waterfall, 15 Dec 1998, *F. Konta, C. Phengklai & S. Khao-Iam 4529* (BKF); **Chiang Rai**: Mae Chan, 20 Jan 1981, *Y. Paisooksantivatana y492-81* (BK, CMUB, KKU); ibid., 1 Dec 2008, *M. Norsaengsri 4470* (QBG); Mae Sai, Pong Pha, Ban Nam Cham, 2 Dec 2008, *M. Norsaengsri 4477* (QBG); Mueang Chiang Rai, Ban Pa Rai, 24 Jan 1981, *Y. Paisooksantivatana 561-81* (BK); **Chon Buri**: Ban Bueng, 3 Jun 1989, *Y. Paisooksantivathana 2327-89* (BK); Si Racha, Thung Sukhla, 8 Dec 2003, *J. F. Maxwell 03-482* (CMUB); **Kamphaeng Phet**: Pang Sila Thong, 13 Nov 2007, *M. Norsaengsri 2986* (QBG)]; **Kanchanaburi**: Thong Pha Phum, 15 Nov 1971, *C. F. van Beusekom, C. Phengklai, R. Geesink & B. Wongwan 3788* (BKF, C, K, P); Thong Pha Phum, Huai Khayeng, Bueng Nam Thip, 3 Dec 2003, *S. Sirimongkol 67* (BKF); Thong Pha Phum, Pilok, 23 Oct 2004, *S. Sirimongkol 164* (BKF); **Khon Kaen**: Nam Nao, 10 Mar 1979, *P. J. O’Corner & C. Niyomdham 15724* (AAU, BKF); Phu Wiang, 21 Feb 1993, *P. Chantaranothai, D. Middleton, J. Parnell & D. Simpson 826* (K, KKU); Phu Wiang, Khok Phu Ta Ka, 30 Nov 2003, *C. Jaroenchai 37* (KKU); Waeng Yai, 26 Dec 2007, *M. Norsaengsri 3255* (AAU, CMUB, QBG); Waeng Yai, Ban Khao Nai, 7 Nov 2008, *M. Norsaengsri 4384* (QBG); **Krabi**: Ao Ma Ya, 24 Nov 1998, *C. Niyomdham 5646* (BKF); Khlong Thom, Khlong Phon, 29 Nov 1986, *J. Supapol 288* (PSU); Khlong Thom, Khlong Thom Tai, 29 Oct 2011, *P. Wessapak 196, 197* (BK, BKF)]; **Lampang**: Doi Luang, 12 Dec 1998, *O. Petrmitr 400* (BKF, CMUB); **Loei**: Phu Ruea, 6 Dec 2004, *C. Jaroenchai 133* (KKU); ibid., 27 Nov 2005, *C. Jaroenchai 163* (KKU), ibid., 12 Nov 2005, *C. Jaroenchai 210* (KKU), ibid., 13 Nov 2005, *C. Jaroenchai 228* (KKU); ibid., 27 Nov 2005, *C. Jaroenchai 245* (KKU); Wang Saphung, 26 May 2009, *M. Norsaengsri & W. La-ongsri 5560* (QBG)]; **Nakhon Nayok**: Khao Yai, Hin Tung, 23 Jan 2002, *J. F. Maxwell 02-35* (BKF, CMUB)]; **Nakhon Pathom**: Phutthamonthon, Salaya, 22 Nov 2002, *J. F. Maxwell 02-418* (BKF, CMUB); **Nakhon Ratchasima**: Khao Yai, 23 Oct 1970, *T. Smitinand 11040* (BKF); Pak Chong, 14 Jan 1965, *Umpai 173* (BK); Pak Thong Chai, Nov 1970, *Ch. Charoenpol, K. Larsen & E. Warncke 4535* (C); Sakaerat, 4 Dec 1983, *N. Fukuoka & M. Ito T-35013* (BKF); **Nakhon Si Thammarat**: Tha Yang, Thung Yai, 2 Nov 2011, *P. Wessapak 201* (BK); Thung Song, 18 Dec 1965, *J. Sadakorn 2* (BK); **Nan**: Tham Sa Koen, 30 Nov 2011, *W. La-ongsri, M. Norsaengsri, P. Panyachan, P. Tatiya & S. Satatha 2002* (QBG, PSU); **Phatthalung**: Phanang Tung, Khuan Khanun, 2 Dec 2012, *P. Na Sawat 2* (PSU); **Phitsanulok**: Thung Salaeng Luang, 25 Jul 1973, *G. Murata, N. Fukuoka & C. Phengklai T-17144* (BKF); ibid., 21 Oct 1984, *G. Murata, C. Phengklai, S. Mitsuta, T. Yahara, H. Nagamasu & N. Nantasan T-38399* (BKF)]; **Phetchabun**: Nam Nao, 25 Oct 2001, *S. Laegaard & M. Norsaengsri 21787* (AAU, BKF, QBG); **Phrae**: Song, Mae Yom, 10 Nov 1991, *J. F. Maxwell 91-1003* (AAU); **Phuket**: Thalaeng, Thep Kasattri, Ban Mai Kao, *J. Supapol 100* (PSU); **Prachin Buri**: Kabin Buri, 13 Feb 2009, *M. Norsaengsri 4854* (QBG); **Rayong**: Klaeng, 15 Feb 2009, *M. Norsaengsri 4877, 4891* (QBG); Klaeng, Chak Don, 18 Nov 2008, *P. Wessumritt & M. Norsaengsri 147, 148* (QBG); **Samut Prakan**: Phra Pradaeng, Song Khanong, 25 Mar 2012, *P. Wessapak & C. Ngernsaengsaruay 204* (BK); **Saraburi**: Wang Muang, Kham Phran, 1 Dec 2011, *P. Wessapak 203* (BK); **Satun**: Mueang Satun, Phi Man, 24 Oct 2010, *P. Wessapak & C. Ngernsaengsaruay 154* (BK, BKF); **Songkhla**: Hat Yai, 2 Feb 1979, *G. Congdon 247* (AAU, PSU); Hat Yai, Ban Nong Bua, 3 Jul 2012, *S. Aya 26* (PSU); Hat Yai, Khao Kho Hong, 24 Oct 2010, *P. Wessapak & C. Ngernsaengsaruay 151* (BK, BKF); Khao Phra, 28 May 2001, *A. Boonprom 1* (PSU); Na Mhom, Klong Rhang, 23 Nov 2016, *P. Wessapak, C. Ngernsaengsaruay, N. Meeprom & W. Boonthasak 344, 345* (BK);Rattaphum, Ban Khao Rak Kiat, 15 Oct 2012, *K. Jamnongjit 27* (PSU); Sadao, Samnak Taeo, 9 Jan 2014, *H. Soh 36* (PSU); **Surat Thani**: Chaiya, Pak Mak, 2 Nov 2011, *P. Wessapak 198* (BK); Chaiya, Pa We, 2 Nov 2011, *P. Wessapak 199* (BK, BKF); **Trang**: Sikao, Mai Fat, Ban Khuan Hen Le, 27 Oct 2011, *P. Wessapak 195* (BK, BKF); **Trat**: Ko Chang, Ban Khlong Son, 22 Mar 2001, *K. Chayamarit, T. Wongprasert, R. Pooma, V. Chaemchumroon, K. Pattarahirankanok & M. Newman 2735* (BKF)]; **Ubon Ratchathani**: Phu Chong Na Yoi, Phalan Kong Kwian, 6 Nov 2010, *P. Wessapak, Y. Buangam & W. Sareemongkonnimit 159* (BK)]; **Udon Thani**: Kumphawapi, Huai Koeng, 4 Dec 2008, *M. Norsaengsri 4582* (QBG).

#### Notes.

This species was introduced to Thailand from India and Philippines as a fodder grass. At present, it has become a major weed and naturalised throughout the country.

Previously, this species was identified as *Pennisetumpolystachion* (L.) Schult. The name was established by J. A. Schultes in 1824, based on the basionym *Panicumpolystachion* L. After the genera were combined, based on the molecular phylogenetic studies (Donadío et al. 2009; Chemisquy et al. 2010), *Pennisetum* was merged into *Cenchrus*. The name of this species in *Cenchrus* is *Cenchruspolystachios* (L.) Morrone coined by Morrone in Chemisquy et al. (2010). Turner et al. (2019) reported that the lectotypification of the basionym *Panicumpolystachion* L. by Merrill in 1917 was based on the plate cited by Linnaeus, which is identified as *Setariaflava* (Nees) Kunth. (now treated as a synonym of *Setariaparviflora* (Poir.) Kerguélen). Therefore, the name of *Cenchrus*, based on *Panicumpolystachion* L., is not correct. The correct name of this species is considered as *Cenchrussetosus* Sw., which has the next priority.

## Supplementary Material

XML Treatment for Cenchrus

XML Treatment for Cenchrus brownii

XML Treatment for Cenchrus ciliaris

XML Treatment for Cenchrus clandestinus

XML Treatment for Cenchrus echinatus

XML Treatment for Cenchrus pedicellatus

XML Treatment for Cenchrus purpureus

XML Treatment for Cenchrus setosus

## ﻿Additional information

### Author ORCIDs

Chatchai Ngernsaengsaruay https://orcid.org/0000-0002-7131-976X

Suthee Duangjai https://orcid.org/0000-0002-1490-759X

### Data availability

All of the data that support the findings of this study are available in the main text.
